# Fine‐Tuning 2D Heterogeneous Channels for Charge‐Lock Enhanced Lithium Separation from Brine

**DOI:** 10.1002/advs.202406535

**Published:** 2024-09-05

**Authors:** Yaxin Hao, Xin Liu, Yaoling Zhang, Xin Zhang, Zhan Li, Ximeng Chen

**Affiliations:** ^1^ MOE Frontiers Science Center for Rare Isotopes Lanzhou University Lanzhou 730000 China; ^2^ School of Nuclear Science and Technology Lanzhou University Lanzhou 730000 P. R. China; ^3^ Institute of National Nuclear Industry Lanzhou University Lanzhou 730000 P. R. China; ^4^ Key Laboratory of Green and High‐end Utilization of Salt Lake Resources Qinghai Engineering and Technology Research Center of Comprehensive Utilization of Salt Lake Resources Qinghai Institute of Salt Lakes Chinese Academy of Sciences Xining 810008 P. R. China; ^5^ Key Laboratory of Comprehensive and Highly Efficient Utilization of Salt Lake Resources Qinghai Institute of Salt Lakes Chinese Academy of Sciences Xining 810008 P. R. China; ^6^ School of Chemistry and Chemical Engineering Qinghai Minzu University Xining 810007 P. R. China

**Keywords:** 2D membrane, charge‐lock, fine‐tuning heterogeneous channels, real brine separation

## Abstract

The extraction of lithium (Li) from complex brines presents significant challenges due to the interference of competing ions, particularly magnesium (Mg^2^⁺), which complicates the selective separation process. Herein, a strategy is introduced employing charge‐lock enhanced 2D heterogeneous channels for the rapid and selective uptake of Li⁺. This approach integrates porous ZnFe_2_O_4_/ZnO nanosheets into Ag^+^‐modulated sub‐nanometer interlayer channels, forming channels optimized for Li⁺ extraction. The novelty lies in the charge‐lock mechanism, which selectively captures Mg^2^⁺ ions, thereby facilitating the effective separation of Li from Mg. This mechanism is driven by a charge transfer during the formation of ZnFe_2_O_4_/ZnO, rendering O atoms in Fe‐O bonds more negatively charged. These negative charges strongly interact with the high charge density of Mg^2^⁺ ions, enabling the charge‐locking mechanism and the targeted capture of Mg^2^⁺. Optimization with Ag⁺ further improves interlayer spacing, increasing ion transport rates and addressing the swelling issue typical of 2D membranes. The resultant membrane showcases high water flux (44.37 L m⁻^2^ h⁻¹ bar⁻¹) and an impressive 99.8% rejection of Mg^2^⁺ in real brine conditions, achieving a Li⁺/Mg^2^⁺ selectivity of 59.3, surpassing existing brine separation membranes. Additionally, this membrane demonstrates superior cyclic stability, highlighting its high potential for industrial applications.

## Graphical abstract

A new strategy for lithium extraction from complex brines uses Charge‐lock enhanced 2D channels made from porous ZnFe_2_O_4_/ZnO nanosheets. This approach selectively captures Mg^2+^ ions through a charge transfer mechanism, improving Li^+^/Mg^2+^ separation. Optimized with Ag^+^, the membrane offers high water flux, 99.8% Mg^2+^ rejection, and superior cyclic stability, making it ideal for industrial use.

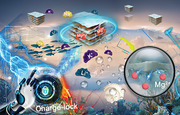

## 1 Introduction

Lithium (Li) stands as a critical element in contemporary technological advancements owing to its indispensability in various sectors such as energy storage, ceramics, and nuclear applications.^[^ ^1^, ^2^ ^]^ The significance of lithium extraction from Salt Lake brines, where ≈70% of terrestrial lithium resources are found, cannot be overstated.^[^ ^3^, ^4^ ^]^ However, the process of extracting Li from such complex brines poses multifaceted challenges, primarily due to the presence of competing ions like magnesium (Mg^2+^), which closely resemble lithium in size, complicating the selective extraction of Li^+^ ions.^[^ ^5^, ^6^, ^7^ ^]^

In this context, membrane separation technology emerges as a pivotal strategy for Li extraction from salt lakes, offering the promise of enhanced efficiency, energy conservation, and environmental sustainability compared to conventional methods such as adsorption, precipitation, and solvent extraction.^[^ ^8^, ^9^ ^]^ Among membrane materials, nanofiltration membrane is widely used due to its high separation efficiency and low energy consumption. However, when treating saline lake water with a high Mg/Li ratio, the accumulation of divalent and higher ions creates a chemical potential difference, necessitating an increase in operating pressure and consequently reducing the separation efficiency.^[^ ^4^, ^10^ ^]^ Therefore, nanofiltration membranes are more suitable for treating brine with a magnesium‐lithium ratio below 30, while brine with a high magnesium‐lithium ratio requires pre‐dilution.^[^ ^11^ ^]^ Additionally, the complex composition of the brine necessitates careful handling to prevent membrane fouling and module blockage.^[^ ^12^ ^]^ Graphene oxide (GO) membranes have garnered significant attention due to their unique structural advantages, including resistance to clogging and promotion of permeability.^[^ ^13^, ^14^, ^15^ ^]^ Despite the potential of GO membranes, their application in Li extraction from salt lakes encounters significant hurdles. Assembling 2D GO into layer‐flow membranes with sub‐nanometer layer spacing offers great opportunities for rapid, large‐scale, and selective transport of ions/molecules.^[^ ^16^, ^17^ ^]^ Challenges such as sub‐nanometer interlayer restacking and swelling hinder the achievement of desired selectivity and structural integrity, thus impeding efficient Li separation.^[^ ^18^, ^19^, ^20^ ^]^

Inspired by advancements in electronic locking mechanisms, we propose a groundbreaking approach that capitalizes on the concept of charge‐lock for lithium separation from brine.^[^ ^21^, ^22^ ^]^ The charge‐lock achieved by applying the principle of fast charge recognition and control.^[^ ^23^ ^]^ Our innovative strategy involves the design and development of a novel material capable of serving as a charge‐lock membrane for lithium separation from complex brines. By integrating the charge‐lock concept with 2D sub‐nanometer heterogeneous channels and tunable spacing within the membrane structure, we introduce a transformative separation strategy that promises unparalleled efficiency and selectivity in lithium extraction from brine.^[^ ^24^ ^]^ The interlayer‐constructed charge‐locking mechanism offers significant advantages over the conventional surface charge repulsion mechanism. It provides greater selectivity in accurately recognizing and trapping specific ions, making it particularly effective for separating ions of similar size but different charge densities. Furthermore, the interlayer channel structure protects the charge lock sites from external environmental influences, significantly enhancing the membrane's stability and durability while alleviating the problem of membrane swelling. The charge interactions within the ion channels reduce impedance, ensuring a balance of high throughput and high selectivity. This pioneering approach not only addresses the limitations of existing membrane technologies but also opens new avenues for efficient and sustainable lithium extraction from salt lake brines.^[^ ^25^, ^26^ ^]^

**Figure 1:**
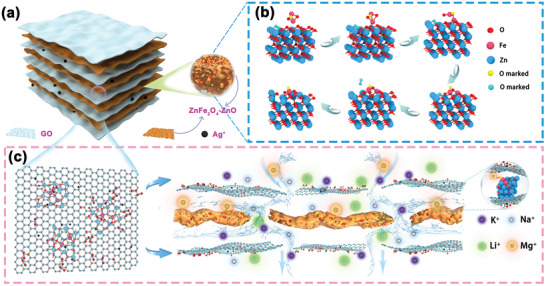
a) Schematic structure of Ag@ZFZ‐GO membranes; b) Molecular dynamics simulation of ZFZ formation process; c) Schematic microscopic ion separation of Ag@ZFZ‐GO membranes.

Hence, we designed a 2D sub‐nanometer GO membrane interlayer channel to combine the location capture capability of charge‐lock with fast transmembrane transport kinetics (**Figure** **1**a). Our approach included the synthesis of porous ZnFe_2_O_4_/ZnO (ZFZ) nanosheets, acting as the fixed‐point negative charge of the charge‐lock, and elucidating its synthesis mechanism in conjunction with molecular dynamics simulations. Additionally, by introducing Ag^+^, we fine‐tuned the sub‐nanometer spacing of GO membrane layers, which lowered the diffusion barrier and enhanced Li^+^ ion penetration (Figure 1c). Experiments and calculations indicated that ZFZ underwent a charge transfer process during its formation, leading to its acquisition of a partial negative charge, and when the higher charge density Mg^2+^ entered the interlayer, it triggered charge‐lock to achieve its location capture, with Li^+^ passing through rapidly. Simultaneously, the interlayer interaction force helps mitigate the swelling effect of GO, thereby enhancing its structural stability. As a result, the prepared Ag@ZFZ‐GO membrane successfully broke the trade‐off between selectivity, permeability, and structural stability, with a water flux as high as 44.37 L m^−2^ h^−1^ bar^−1^, Mg^2+^/Li^+^ separation factor up to 59.3, and maintained great stability after cycling for 240 h (20 times). These results pave the way for cost‐effective ion separation in complex media.

## 2 Synthesis of ZnFe_2_O_4_‐ZnO Nanosheets

Drawing upon foundational theories of charge transfer during oxide formation, our research presents a novel porous ZnFe_2_O_4_‐ZnO (ZFZ) nanosheet, emphasizing the significance of charge dynamics in the oxide framework.^[^ ^27^, ^28^ ^]^ Through the application of density‐functional theory (DFT) and molecular dynamics simulations, we have delineated the synthesis process of ZFZ nanosheets, further illustrated through supplementary animations (MD_0.mp4, MD_1.mp4).^[^ ^29^, ^30^ ^]^ Our findings highlight a critical interaction between Fe_2_O_3_ and ZnO (101) surfaces at elevated temperatures (500 °C), leading to the detachment of oxygen molecules and the creation of oxygen vacancies (O_V_). This phase transition, characterized by a redistribution of electrons, results in the formation and stabilization of the ZFZ structure, as illustrated in Figure 1b.^[^ ^31^ ^]^

To corroborate our theoretical predictions, we undertook an exhaustive characterization of the synthesized ZFZ nanosheets, focusing on their morphology and chemical composition. Scanning electron microscopy (SEM) analyses, as shown in Figure S1a–c and Figures S2–S4 (Supporting Information), unveiled a significantly smoother and more homogeneous surface morphology of the ZFZ samples compared to standalone ZnO and Fe_2_O_3_, suggesting improved interfacial interaction with the GO membrane and augmented stability. Elemental distribution (EDS) mapping confirmed the homogeneous distribution of Fe, Zn, and O across the ZFZ structure. Further, Transmission Electron Microscopy (TEM) and High‐Resolution TEM (HRTEM) investigations, along with Fast Fourier Transform (FFT) mapping (illustrated in Figure S1d and Figures S5–S7, Supporting Information), validated the successful synthesis of ZFZ. The observed lattice fringes at 0.296 nm and 0.253 nm matched the (220) and (311) crystal planes of ZnFe_2_O_4_, respectively, while 0.247 nm and 0.281 nm fringes corresponded to the ZnO (101) and (100) crystal planes.^[^ ^27^, ^32^ ^]^ X‐ray diffraction (XRD) patterns, detailed in Figure S1e (Supporting Information), confirmed the crystal structure and purity of the synthesized oxides, aligning with standard references for ZnO, Fe_2_O_3_, and ZnFe_2_O_4_ crystallites.^[^ ^33^, ^34^, ^35^ ^]^ Moreover, as shown in Figure S8 (Supporting Information), the main diffraction peaks observed in the experiment and the simulation are consistent, thus validating the accuracy of our established model. Remarkably, both Electron paramagnetic resonance (EPR) and X‐ray photoelectron spectroscopy (XPS) analyses indicated a significant reduction in oxygen vacancies in the ZFZ nanosheets relative to ZnO samples alone. This finding suggests enhanced interfacial bonding and chemical stability stemming from the incorporation of Fe_2_O_3_, with pure Fe_2_O_3_ showing no detectable EPR signal (Figure S1f, Supporting Information).^[^ ^36^, ^37^ ^]^

XPS analysis delineated the O 1s spectra of ZFZ, ZnO, and Fe_2_O_3_ into three distinct peaks corresponding to lattice oxygen (O_L_) at 530.2 eV, O_V_ at 531.5 eV, and chemisorbed oxygen species (O^2−^ or O^2‐^ ion) (O_C_) at 532.7 eV (Figure S1j–k, Supporting Information).^[^ ^38^, ^39^, ^40^ ^]^ These findings, along with their relative abundances detailed in Table S1 (Supporting Information), indicate a decrease in the proportion of O_V_ in ZFZ compared to ZnO, aligning with EPR results. Additionally, the increased levels of O_V_ and O_C_ suggest enhanced reactive sites for ion adsorption on ZFZ, potentially improving interactions with targeted ions and increasing surface charge affinity for oppositely charged species, realizing a charge‐lock mechanism.^[^ ^41^, ^42^ ^]^ High‐resolution XPS spectra for Zn 2p and Fe 2p (Figure S1h–i, Supporting Information) revealed binding energy shifts in ZFZ, suggesting a reduction in oxygen vacancies compared to ZnO and a higher electron density around iron atoms relative to Fe_2_O_3_.^[^ ^37^, ^43^ ^]^ These shifts imply an augmentation in the adsorption capabilities of ZFZ for ions bearing an opposite charge. Thermogravimetric (TGA) analysisconfirmed the good thermal stability of each oxide (Figure S1m, Supporting Information).^[^ ^44^ ^]^ These comprehensive characterisations underline the successful synthesis and stability of ZFZ nanosheets and provide a solid foundation for the construction of 2D graphene oxide sub‐nanochannel membranes with charge‐lock.

## 3 2D Ag@ZFZ‐GO Membrane Interlayer Channels

**Figure 2:**
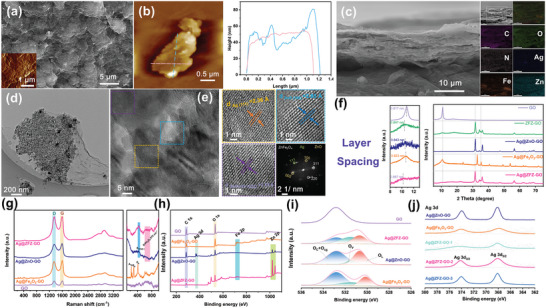
Structural characterization of GO, Ag@ZnO‐GO, Ag@Fe_2_O_3_‐GO and Ag@ZFZ‐GO series membrane materials. a,c) The SEM, b) AFM, and d,e) TEM images of Ag@ZFZ‐GO membrane. f) XRD patterns and amplified XRD patterns of selected areas; g) Raman spectra; h) XPS spectra for survey; i) O 1s spectra; j) Ag 3d spectra.

As depicted in the synthesis schematic (Figure S9, Supporting Information), we engineered sub‐nanometer interlayer channels leveraging the charge‐lock mechanism, capitalizing on the negative charge induced by charge transfer during the formation of ZFZ nanosheets. By introducing Ag^+^, we fine‐tuned the interlayer spacing within the sub‐nanometer domain, aiming to enhance the ion transfer rate. Surface and structural analyses via SEM and Atomic force microscopy (AFM) (**Figure** **2**a–b) revealed that the Ag@ZFZ‐GO membranes possessed uniform surfaces with distinct lamellar structures, and an average nanosheet thickness of approximately 50 nm. Cross‐sectional SEM images (Figure 2c; Figure S10, Supporting Information) showcased a consistent book‐like lamellar stacking across all synthesized 2D GO membranes, with Ag@ZnO‐GO and Ag@Fe_2_O_3_‐GO mirroring the morphologies of their oxide precursors. Notably, interlayer spacing within Ag@ZFZ‐GO membranes expanded with increased Ag^+^ incorporation, highlighted by significant nanoparticle formation in Ag@ZFZ‐GO‐3 membranes (Figure S11, Supporting Information). The successful embedding of ZFZ nanosheets and Ag^+^ into the GO interlayers was confirmed using EDS mapping. The TEM of Ag@ZFZ‐GO membranes (Figure 2d) preserved the porous structure of ZFZ beneath a veneer of ultrathin GO, with HR‐TEM and FFT analyses (Figure 2e) identifying lattice fringes corresponding to Ag, ZFZ, and ZnO crystal planes.^[^ ^45^, ^46^ ^]^ In addition, XRD patterns (Figure S12a, Supporting Information) a notable shift of the GO(001) peak toward a smaller angle following the incorporation of ZFZ, indicative of an increased interlayer distance (the interlayer distance of the Ag@ZFZ‐GO film calculated by the Bragg formula is 8.67 Å).^[^ ^19^, ^45^ ^]^ This alteration in interlayer spacing, not exceeding 2 Å, suggests that Ag^+^ ions play a pivotal role in modulating the sub‐nanometer level interlayers. It is noteworthy that when the Ag^+^ content is too high (Ag@ZFZ‐GO‐3), all the diffraction peaks are significantly shifted, implying that the excess Ag^+^ enters into the lattice structure of ZFZ, consequently changing the charge distribution state of the material.^[^ ^16^, ^47^ ^]^

As shown by Fourier infrared spectroscopy (FTIR) (Figure S12b–c, Supporting Information), the inclusion of Ag^+^ and ZFZ does not modify the characteristic functional groups of GO. The peak at ≈989 cm^−1^ corresponds to metal‐oxygen bond vibrations. Additionally, the intensification of the peak at 911 cm^−1^ with the addition of Ag^+^, aligns with XRD results, suggesting enhanced metal‐oxygen interactions due to Ag^+^ integration.^[^ ^39^, ^40^ ^]^ Raman spectroscopy (Figure 2g; Figure S12d, Supporting Information) further supports these findings, displaying characteristic G‐band (≈1631 cm^−1^) and D‐band (≈1395 cm^−1^) peaks, representative of sp^2^ hybridized carbon structures and lattice defects within the GO membranes.^[^ ^48^, ^49^ ^]^ The Zn‐O characteristic peak appears at 438.0 cm^−1^, and a broad peak at 672 cm^−1^ results from surface phonon scattering of the Zn boundary phonon (2E_2_(M) + A_1_(LO)) after constructing the in‐plane ZFZ structure.^[^ ^50^ ^]^ In addition, the I_D_/I_G_ value of Ag@ZFZ‐GO series membranes increased from 1.77 to 2.25 with the increase of Ag^+^ content, indicating the increase of defective sites in the material after the introduction of Ag^+^, which is favorable for the recognition and transport of ions.

Moreover, the XPS spectra (Figure 2h; Figure S12e, Supporting Information) confirmed the composite's composition, including C, O, Fe, Zn, and Ag elements within the Ag@ZFZ‐GO membranes. High‐resolution XPS (Figure S12f–g and Table S2, Supporting Information) revealed changes in the GO matrix due to Ag^+^ and ZFZ doping: a decrease in C‐O components and an increase in C‐C and C = O components, indicative of reduced GO swelling and enhanced stability.^[^ ^8^, ^9^ ^]^ The O 1s spectrum analysis (Figure 2i; Figure S12h and Table S3, Supporting Information) showed a decrease in O_L_ and an increase in O_V_ and O_C_ + Oxygen‐containing groups (O_C_+O_cg_), with variations in Ag^+^ concentration contributing to increased surface activity and electrostatic interactions to trigger the charge‐lock mechanisms.^[^ ^51^ ^]^ In cases of excess Ag^+^ (Ag@ZFZ‐GO‐3), the O_V_ diminished and the O_C_+O_cg_ surged, likely due to the Ag^+^‐induced surface reconstruction of GO, which reactivated the otherwise inactive functional groups.^[^ ^16^, ^45^ ^]^ Furthermore, in the case of Ag@ZnO‐GO and Ag@Fe_2_O_3_‐GO, compared to ZnO and Fe_2_O_3_ alone, an increase in Ag^+^ addition led to a decrease in both O_L_ and O_V_. This reduction is likely attributed to the weak interfacial interactions between ZnO and Fe_2_O_3_ with GO, which potentially alter the chemical states of the pristine oxides and GO.^[^ ^40^, ^52^ ^]^ The peaks of Fe 2p spectra (Figure S12i–j, Supporting Information) at ≈711.3 eV and ≈725.1 eV belonged to Fe 2p_3/2_ and Fe 2p_1/2_, respectively, and could be further fitted to Fe^3+^ (≈712.8 eV/≈727.4 eV) and Fe^2+^ (≈710.7 eV/≈724.5 eV), with Fe^3+^ content rising as Ag^+^ concentration increases.^[^ ^46^ ^]^ In addition, the Zn 2p spectra (Figure S12k–m, Supporting Information) also showed that the peaks at Zn 2p_3/2_ and Zn 2p_1/2_ shifted to higher binding energies with the increase of Ag^+^ addition, suggesting that the incorporation of Ag^+^ leads to the reduction of oxygen vacancies due to the charge balance and spatial site resistance effects, which verifies the previous analysis of O 1s.^[^ ^43^ ^]^

## 4 Separation Performance of Ions

**Figure 3:**
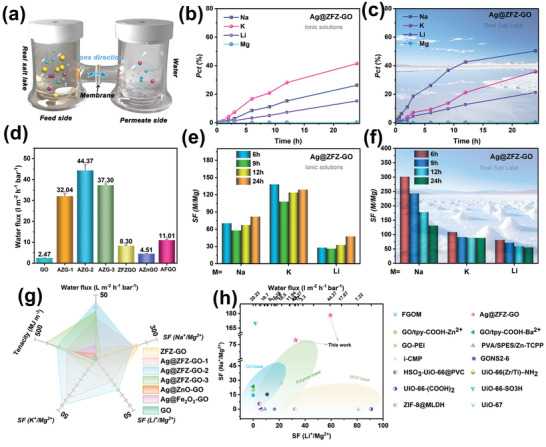
a) Schematic diagram of the permeation separation device; b,c) Permeation kinetic curves of Ag@ZFZ‐GO membrane under ionic solution and real salt lakes as well as e,f) the separation factor of Mg^2+^ from monovalent ions at 6–24 h; d) Water flux test data of the prepared membrane material. g,h) Comparison plots between the properties of the prepared membranes and with the current published literature.

To evaluate the permeability and separation performance of Ag@ZFZ‐GO membranes, we conducted permeation separation experiments under equal concentrations of ionic solutions, simulated salt lakes, and real salt lakes systems (**Figure** **3**a for experimental setups and Tables S4–S6, Supporting Information for ionic concentrations). The experiment setup involved positioning a 2D GO membrane vertically in an H‐glass filter, creating two distinct compartments: the feed compartment (FC), containing the permeate solution, and the receiver compartment (RC), filled with deionized water at various pH levels to act as the driving fluid. The membrane, with an effective contact area of 2.554 cm^2^, separates these compartments. To mitigate concentration polarization effects during permeation experiments, the solution in both compartments was stirred using a magnet at 120 rpm. Furthermore, we calculated the change in ΔP and the corresponding hydrostatic pressure difference ΔP based on the height difference between the water levels in the left and right compartments of the separation unit after 72 h of separation (ΔP = ρgΔh, with ρ representing water density set at 1000 kg m^−^^3^ due to the dilute nature of both solutions). The results showed that ΔP was 52 Pa at 24 h and that the transmembrane osmotic pressure gain decreased as the reaction progressed, eventually reaching equilibrium (Figure S13, Supporting Information).^[^ ^53^ ^]^ Following this setup, we verified the ZFZ charge‐lock mechanism and the effect of Ag^+^ on permeability and selectivity by investigating the permeation kinetics of mixed ionic solutions (Figure 3b–e; Figures S14–S15, Supporting Information). The permeation percentages (pct (%), expressed as the percentage of the concentration of a specific element that permeates from the permeate to the driving fluid, which theoretically can only reach 50%)^[^ ^7^, ^18^ ^]^ followed a sequence of K^+^>Na^+^>Li^+^>Mg^2+^, with Mg^2+^ showing a permeation capacity under 1%, proving its ability to trigger charge locks for the efficient location capture Mg^2+^. Notably, with the addition of Ag^+^, the pct of Mg^2+^ remained low, while the pct of other monovalent ions increased significantly, and the selectivity of Mg^2+^ with other monovalent ions in the Ag@ZFZ‐GO membranes at 12 h were K^+^/Mg^2+^ = 123.7, Na^+^/Mg^2+^ = 67.2, and Li^+^/Mg^2+^ = 32.5. In addition, the highly variable Mg/Li ratios observed in many real salt lakes pose a significant challenge for membrane applications. In order to gain a deeper understanding of the separation mechanism and performance of the prepared series of membranes, salt solution separation experiments with the addition of Mg^2+^ ions (Mg^2+^/Li+ ratio of 50, Figure S15 and Table S5, Supporting Information) were performed. The results showed that the separation effect of Ag@ZFZ‐GO membranes was not significantly reduced even at high Mg/Li ratios. To validate the ion recognition ability of Ag@ZFZ‐GO in real complex systems, we performed separation tests in simulated salt lakes as well as real salt lakes. As shown in Figure 3c–f and Figure S16, the pct of Mg^2+^ in Ag@ZFZ‐GO membrane remained under 1% after 24 h, maintaining the selectivity order Na^+^>K^+^>Li^+^>Mg^2+^, proving its excellent Mg^2+^ selectivity even under the real system. Interestingly, the Na^+^ pct from real salt lakes notably increased compared to synthetic ionic solutions, attributable to the higher Na^+^ concentration in real salt lakes. The comparative analysis of permeation kinetics under real salt lakes (Figure S16, Supporting Information) revealed that Ag@ZFZ‐GO membranes exhibited superior selectivity —K^+^/Mg^2+^ = 89.1, Na^+^/Mg^2+^ = 178.2, and Li^+^/Mg^2+^ = 59.3—outperforming Ag@ZnO‐GO, Ag@Fe_2_O_3_‐GO, and pure GO membranes. Excellent compared to the currently published brine separation membranes as well (Figure 3g–h; Table S7, Supporting Information). Furthermore, Ag@ZnO‐GO and Ag@Fe_2_O_3_‐GO membranes (Figure S17, Supporting Information) exhibited limited Mg^2+^ selectivity, which was attributed to variations in charge density and distribution, with stronger electrostatic interactions of ZFZ with Mg^2+^ triggering the charge‐lock mechanism, but not ZnO and Fe_2_O_3_.^[^ ^4^, ^25^ ^]^

Furthermore, the addition of Ag^+^ significantly enhanced the separation efficiency and water flux of Ag@ZFZ‐GO membranes. The insertion of ZFZ led to a fourfold increase in the water flux of ZFZ‐GO membranes due to expanded layer spacing and enhanced hydrophilicity, providing more channels for water molecule transport. The addition of Ag^+^ further increased interlayer spacing, defect sites, and surface roughness, resulting in a water flux of 44.37 L m^−2^ h^−1^ bar^−1^ for Ag@ZFZ‐GO‐2 membranes, which is 22 times higher than that of GO membranes, as confirmed by contact angle and membrane surface roughness measurements (Figure 3d; Figures S18a–g and S19, Supporting Information). However, additional increments of Ag^+^ resulted in a decrease in the water flux of Ag@ZFZ‐GO‐3 membranes, potentially due to the formation of silver particles or aggregates within the pores or on the surface of the membranes.^[^ ^37^, ^54^ ^]^ Permeation kinetics in real salt lakes corroborated these findings, with Ag^+^ enhancing the pct of monovalent ions while preserving the selectivity for Mg^2+^ (Figure S20, Supporting Information). The reduced selectivity observed in Ag@ZFZ‐GO‐3 membranes was linked to excessive doping of Ag^+^ into the crystal lattice of ZFZ, altering the surface charge density and affecting the electrostatic attraction to Mg^2+^. Adjustment of the pH of the driving solution had minimal impact on permeability but reduced separation efficiency (Figure S21, Supporting Information), with the optimal separation achieved when using deionized water (pH≈7) as the driving solution.^[^ ^8^, ^49^ ^]^ Furthermore, commercial nanofiltration membranes are a well‐established technology for salt lake separations, but often the membranes are compacted to reduce selectivity and permeability as the pressure increases.^[^ ^55^ ^]^ Therefore, additional experiments were conducted to assess the membrane materials for commercial nanofiltration applications. Under nanofiltration conditions at 5 bar pressure and 25 °C (Figure S22a, Supporting Information), the Ag@ZFZ‐GO membranes exhibited significant rejection rates, with 98.39% rejection of equal concentration ionic solutions and 99.21% rejection of real salt lake water,. outperforming most of the nanofiltration membranes that have been reported to date. XRD analysis (Figure S22b, Supporting Information) revealed that the main diffraction peaks of Ag@ZFZ‐GO membrane remained unchanged before and after nanofiltration, with the (001) peak of graphene oxide showing a slight rightward shift, indicating minor structural compression under pressure. SEM tests (Figure S22c, Supporting Information) demonstrated that both the surface and cross‐section of Ag@ZFZ‐GO membrane maintained exceptional stability throughout the experiments, with no apparent cracks or structural collapse. These findings underscore the exceptional separation performance of the Ag@ZFZ‐GO under rigorous operational conditions.

Given the critical importance of stability in the commercialization of 2D membranes, the 2D GO membranes underwent rigorous tensile stress‐strain, immersion, and cycling tests. Figures S18h–i and S23a (Supporting Information) demonstrated that Ag@ZFZ‐GO membranes could regain their original shape post 180° folding, exhibiting a tenacity of 329.2 MJ m^−3^, superior to Ag@ZnO‐GO and Ag@Fe_2_O_3_‐GO membranes. After 15 days immersed in a pH≈7 aqueous solutions, all membranes, with the exception of the Ag@Fe_2_O_3_‐GO membrane, preserved their original appearance. XRD analysis revealed structural stability; the (001) diffraction peak of GO membranes shifted significantly, while Ag@ZFZ‐GO materials exhibited minimal changes, and ZFZ's major diffraction peaks remained unchanged (Figure S24, Supporting Information). Moreover, Ag@ZFZ‐GO membranes underwent 20 real salt lakes separation trials (each separation was followed by washing of organic contaminants using 0.1 M NaOH solution, treatment of inorganic deposits with 0.1 M HCl solution, and then thorough rinsing with deionized water), maintaining their performance and selectivity without significant loss after 240 h (Figure S23b,c, Supporting Information). The XRD patterns before and after separation (Figure S23d, Supporting Information) and SEM images (Figure S25, Supporting Information) confirmed the stability of the Ag@ZFZ‐GO membrane. No obvious contaminants were observed on the membrane surface before and after separation, and the morphology before separation was retained. Additionally, the main diffraction peaks did not change significantly, highlighting that the membrane is suitable for industrial‐scale production and effectively overcomes challenges related to membrane clogging and regeneration capacity.

## 5 Investigation of Separation Mechanisms

The experimental data revealed that the permeation rate sequence of ions across the synthesized 2D membranes corresponds to their hydration diameter order. The hydration diameters of the separated ions K^+^ (6.62 Å), Na^+^ (7.16 Å), Li^+^ (7.64 Å) and Mg^2+^ (8.56 Å) were lower than the interlayer distance of the Ag@ZFZ‐GO membrane (8.67 Å).^[^ ^12^, ^56^ ^]^ As the ions diffuse within the limited interlayer spacing, their hydration shells undergo partial dehydration, further reducing the effective sizes.^[^ ^57^, ^58^ ^]^ Therefore, size exclusion is not sufficient for the sub‐nanometer GO membranes to block Mg^2+^ transport. In addition, Zeta potential measurements (Figure S26a, Supporting Information) indicate that the membrane surface is negatively charged under the experimental conditions. The introduction of ZnFe₂O₄‐ZnO nanosheets alters the overall surface charge state due to interactions with functional groups (e.g., hydroxyl, carboxyl) on the chemographene, as well as the intrinsic charge properties of the ZnFe₂O₄‐ZnO nanosheets. The potential gradually shifts to a more positive value as the Ag^+^ concentration increases. This shift results from the electrostatic interactions between Ag^+^ and GO, leading to an overall enhancement of the positive zeta potential, with the addition of Ag^+^ primarily facilitating faster penetration of the monovalent cations. Furthermore, the surface positive charge of the Ag@ZFZ‐GO membrane increases after the separation of different ions (Figure S26b, Supporting Information), with Mg^2^^+^ exhibiting a particularly pronounced positive trend. This observation suggests that the Ag@ZFZ‐GO membrane preferentially adsorbs Mg^2^^+^, achieving a charge‐lock mechanism. Importantly, this enhanced positive charge upon Mg^2^^+^ ion adsorption increases the repulsive force on Mg^2^^+^ ions, thereby enhancing the membrane's selectivity for Mg^2^^+^ relative to other ions, such as Li^+^.^[^ ^58^ ^]^ Additionally, to verify the stability of Ag^+^ ions and their effect on membrane charge distribution, we performed XPS analysis before and after the separation experiments (Figure S23i– m, Supporting Information). The results showed that the Ag^+^ ions remained stable within the membrane structure throughout the separation process, with the nearly constant position of the Ag peaks indicating that the Ag^+^ ions retained their binding environment and charge distribution state during separation, without significant migration or chemical transformation.

**Figure 4:**
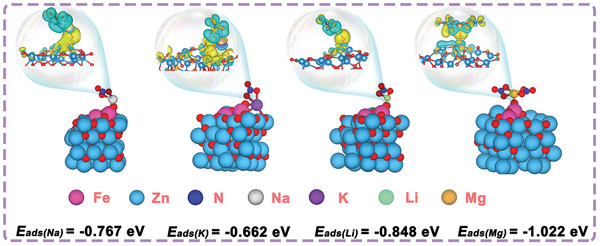
Schematic diagram of the structure after optimization bound between Na^+^, K^+^, Li^+^, and Mg^2+^, respectively, as well as plots of the charge density difference and adsorption energy data (the yellow and green isosurfaces stand for the areas of increased charge and decreased charge, respectively).

To further elaborate the separation mechanism of Ag@ZFZ‐GO membranes, the DFT calculations were performed. Energy minimization optimized coordination adsorption of Na^+^, K^+^, Li^+^, and Mg^2+^ ions on the model surface. Adsorption energies for marked oxygen atoms on the surface of ZFZ interacting with mono and divalent ions were calculated, employing the structures after optimization from MD simulations. Calculated adsorption energies showed that the interaction of Mg with O (−1.02 eV = −98.5 kJ mol^−1^) was significantly stronger than interactions of monovalent ions (*E*_ads(Li)_ = −0.85 eV = −81.97 kJ mol^−1^, *E*_ads(K)_ = −0.66 eV = −63.65 kJ mol^−1^, *E*_ads(Na)_ = −0.77 eV = −74.26 kJ mol^−1^), indicating a stable adsorption of Mg^2+^ onto the surface of ZFZ, which facilitates specific Mg^2+^ recognition and subsequent Li extraction.^[^ ^58^, ^59^ ^]^ Charge density differences, as depicted in **Figure** **4**, qualitatively illustrate variations in atomic surface charge post‐interaction, with the interaction involving Mg showing more pronounced yellow regions on marked oxygen atoms on the surface of ZFZ, indicating stronger electrostatic interactions and a higher affinity for Mg^2+^ adsorption compared to monovalent ions, triggering the charge‐lock mechanism more readily to achieve an efficient separation of Li^+^/Mg^2+^.^[^ ^34^, ^60^ ^]^ XPS analysis further substantiated theoretical calculations; K 1s and Li 1s peaks exhibited no significant features, Na 1s peaks at 1071.5 eV corresponded to sodium compounds, and Mg 1s peaks at 1304.5 eV confirmed the presence of Mg‐O bonds, supporting DFT results (Figure S23e–h, Supporting Information).^[^ ^61^, ^62^ ^]^ To verify this, immersion experiments and Arrhenius tests of various ions were performed using Ag@ZFZ‐GO membranes at different temperatures (Figure S27, Supporting Information). The results demonstrated that immersion in solutions containing different ions led to changes in layer spacing, with Mg^2+^ showing the most significant leftward shift in layer spacing. This indicates stronger interaction between Mg^2+^ ions and our prepared materials, suggesting greater propensity for adsorption within the interlayer space. Moreover, the observed shifts in interlayer spacing in mixed ion solutions resembled those in single Mg^2+^ solutions, further supporting Mg^2+^’s robust adsorption properties.^[^ ^12^, ^63^ ^]^

Arrhenius activation energies (Ea) for Na^+^ (1.94 kcal mol^−1^, 8.12 kJ mol^−1^), K^+^ (1.75 kcal mol^−1^, 7.32 kJ mol^−1^), Li^+^ (2.14 kcal mol^−1^, 8.95 kJ mol^−1^), and Mg^2+^ (3.09 kcal mol^−1^, 12.93 kJ mol^−1^) were found to be lower than their respective adsorption energies. This suggests that the energy required for ion movement within the membrane material is not substantial, likely due to partial dehydration of ions upon entering the interlayer. This reduction in energy barrier facilitates ion transport, highlighting that the separation process is primarily driven by adsorption.^[^ ^26^, ^64^ ^]^ The strong adsorption of ions on both the membrane surface and within the membrane (with significant negative adsorption energy) serves as the primary force for separation. The magnitude of adsorption energy dictates the order of preferential ion adsorption on the membrane, thereby achieving effective separation.

In summary, we engineered sub‐nanometer channels using ZnFe_2_O_4_/ZnO nanosheets intercalated within GO interlayers to trigger a charge‐lock mechanism for selective recognition of Mg^2+^ during Lithium harvesting from complex brines. Moreover, inserting Ag^+^ allowed fine‐tuning of the sub‐nanolayer spacing without compromising membrane selectivity, instead enhancing the stability and permeability. This approach successfully reconciles the notorious trade‐off between selectivity, permeability and stability in conventional 2D membranes by enabling charge density‐dependent ion sieving. Additionally, the combination of experimental and computational studies offered insights into the separation mechanisms, highlighting the vital roles of surface chemistry, charge distribution, and local chemical milieu. Our findings provide new perspectives on deploying 2D sub‐nanomaterials for separation applications, and pave the way toward designing next‐generation ion separation and desalination membranes with superb efficiency.

## Conflict of Interest

The authors declare no conflict of interest.

## Author Contributions

Y.H., X.Z., and Z.L. conceived the idea. Y.H. and Z.L. designed the experiments, analyzed the data, and wrote the manuscript. Y. H. synthesized and characterized the membranes. Xin Zhang performed density‐functional theory simulations. X.Z., Z.L., X. L., Y.Z., and X.C. discussed the results and commented on the manuscript.

## Supporting information

Supporting Information

## Data Availability

The data that support the findings of this study are available in the supplementary material of this article.
