# Supplementary material for: Fine‐Tuning 2D Heterogeneous Channels for Charge‐Lock Enhanced Lithium Separation from Brine
Source: Adv Sci (Weinh). 2024 Sep 5;11(41):2406535. doi: 10.1002/advs.202406535 (PMC11538673; doi:10.1002/advs.202406535)
Supplement: Supplementary file 1 — Supporting Information [file ADVS-11-2406535-s001.docx]

Fine-tuning two-dimensional heterogeneous channels for charge-lock enhanced lithium separation from brine

Yaxin Hao^1,2,3#^, Xin Liu^4^, Yaoling Zhang^5^, Xin Zhang^1,2,3^, Zhan Li^1,2,3,6^[[1]](#footnote-1)^^, Ximeng Chen^1,2,3^

*^1^MOE Frontiers Science Center for Rare Isotopes, Lanzhou University, Lanzhou 730000, China.*

*^2^School of Nuclear Science and Technology, Lanzhou University, Lanzhou 730000, P. R. China.*

*^3^Institute of National Nuclear Industry, Lanzhou University, Lanzhou 730000, P. R. China.*

*^4^Key Laboratory of Green and High-end Utilization of Salt Lake Resources, Qinghai Engineering and Technology Research Center of Comprehensive Utilization of Salt Lake Resources, Qinghai Institute of Salt Lakes, Chinese Academy of Sciences, Xining 810008, P. R. China.*

*^5^Key Laboratory of Comprehensive and Highly Efficient Utilization of Salt Lake Resources, Qinghai Institute of Salt Lakes, Chinese Academy of Sciences, Xining 810008, P. R. China*

*^6^School of Chemistry and Chemical Engineering, Qinghai Minzu University, Xining 810007, P. R. China.*

Content

[Material characterization 3](#_Toc157695115)

[Methods 4](#_Toc157695116)

[Theoretic calculation 8](#_Toc157695117)

[Figures 10](#_Toc157695118)

[Tables 29](#_Toc157695119)

[References 38](#_Toc157695120)

# Material characterization

Surface morphological characteristics of the synthesized materials were meticulously characterized using Thermo Scientific Apreo S Scanning Electron Microscope (SEM), complemented by detailed elemental mapping analysis. Micro-nanostructural features and chemical composition determinations were performed utilizing FEI Talos G2 F200X Transmission Electron Microscope (TEM), integrated with Energy Dispersive X-ray Spectroscopy (EDX) capabilities. X-ray Photoelectron Spectroscopy (XPS) analysis was conducted employing Kratos AXIS Ultra DLD, meticulously calibrated with a 284.6 eV C 1s peak, to elucidate the chemical structure and coordination environments. Furthermore, comprehensive X-ray Diffraction (XRD) analyses were performed employing Rigaku Ultima IV Diffractometer, featuring Cu Kα radiation (wavelength 1.541871 Å). Electron Paramagnetic Resonance (EPR) Spectroscopy measurements were characterized with a Bruker ER200DSRC10/12 Spectrometer. Raman Spectral analysis was carried out using Rigaku Corporation instrument, while Fourier-Transform Infrared (FTIR) Spectra were acquired utilizing NICOLET's NEXUS 670. Thermogravimetric Analyzer (TGA) are obtained on a Thermostep (ELTRA, Germany). Atomic force microscopy (AFM, Bruker Dimension Icon, USA) was used to characterize the morphology and roughness of the membranes.Ionic solution concentrations were quantitatively assessed employing Jena PQ9000 Inductively Coupled Plasma Emission Spectrometer (ICP-OES).

# Methods

**Materials**

The following reagents were acquired: 200 mesh graphite powder, ferrous chloride tetrahydrate (FeCl_2_·4H_2_O, >99.5%), urea (CO(NH_2_)_2_, >99.5%), sodium nitrate, (NaNO_3_, >99.0%), silver nitratezinc (AgNO_3_, >99.8%), zinc acetate dihydrate (C_4_H_6_O_4_Zn·2H_2_O, >99.0%), and sodium borohydride (NaBH_4_, >98.0%) from Aladdin Biochemical Technology Co., Ltd.; hydrogen peroxide (H_2_O_2_, 30wt%) from Guangfu Chemical Reagent Factory, Tianjin; sulfuric acid (H_2_SO_4_, ≥98.3%) and hydrochloric acid (HCl,≥99.7%) from Chengdu Kelong Chemical Co.; potassium permanganate (KMnO4, >99.5%) from Tianjin Kemeo Chemical Reagent Co., Ltd.; sodium chloride (NaCl, >99.0%), potassium chloride (KCl, >99.0%), magnesium chloride hexahydrate (MgCl_2_·6H_2_O, >99.0%), and lithium chloride (LiCl, >99.0%) from Tianjin Damao Chemical Reagent Factory; and PES polyethersulfone microporous membrane from Haiyan New Oriental Plastic Chemical Technology Co., Ltd.

**Fabrication of Graphene Oxide (GO)**

Graphene oxide was synthesized using the modified Hummers method^[1]^. Initially, 5 g of graphite powder and 5 g of NaNO_3_ were mixed in a 500 ml three-necked flask. Then, 200 ml of concentrated sulfuric acid was gradually added and the mixture was stirred in an ice bath for 1 h. Next, 20 g of KMnO_4_ was added gradually, the mixture was heated to 38°C, and stirred at 200 rpm for 26 h. After that, 250 ml of deionized water was added dropwise; the solution was heated first to 84°C and then allowed to cool to room temperature. The mixture was then transferred to a beaker containing 500 ml of deionized water, and 30% H_2_O_2_ was added dropwise until no further reaction was observed. Finally, the solution was washed three times with 1 L of 10% HCl and repeatedly with deionized water until neutral, and stored at a low temperature for later use.

**Fabrication of ZnO, Fe_2_O_3_ and ZnFe_2_O_4_-ZnO (ZFZ) porous nanosheets**

2.1951 g of C_4_H_6_O_4_Zn·2H_2_O and 2.1021 g of CO(NH_2_)_2_ were weighed and added to a 50 ml beaker, followed by the addition of 20 ml of deionized water to dissolve the mixture, which was then stirred at 800 rpm for 2 h. In a separate solution, 318.09 mg of FeCl_2_·4H_2_O was dissolved in 50 ml of water, to which 37.83 mg of NaBH_4_ was added, and stirred at 800 rpm for 2 h. The zinc acetate-urea solution was then added dropwise to the FeCl_2_ solution and stirred for 1.5 h. This mixed solution was transferred to a PTFE-lined autoclave reactor and heated at 100°C for 12 h. After completion, the resultant powder was washed thrice with deionized water, dried at 80 °C overnight, and then calcined under an O_2_ atmosphere at 500°C for 24 h to yield an orange-colored powder, designated as ZnFe_2_O_4_-ZnO (ZFZ). The preparation of ZnO nanosheets and Fe_2_O_3_ nanoparticles followed a similar procedure but without mixing the components.

**Fabrication of Ag@ZnO-GO, Ag@Fe_2_O_3_-GO, GO and Ag@ZFZ-GO series membrane**

20 mg of ZFZ-ZnO powder was weighed into a 50 ml beaker, followed by the addition of 20 ml of deionized water. The mixture underwent ultrasonic dispersion and was stirred at 800 rpm. Then, 1.25 mL of 8 g/L GO dispersion was added and stirred for 0.5 hours, followed by the addition of 100 µL of 0.01 M AgNO_3_ solution and stirred for 1 hour. Ag@ZFZ-GO membranes were then prepared by suction filtration using a PES polyethersulfone microporous membrane. ZFZ-GO membranes were similarly prepared, omitting the AgNO_3_ solution. The Ag@ZFZ-GO-1, Ag@ZFZ-GO-2, and Ag@ZFZ-GO-3 variants were prepared by the amount of AgNO_3_ added: 50 µL, 100 µL, and 200 µL, respectively. For Ag@ZnO-GO and Ag@Fe_2_O_3_-GO membranes, the process was identical, except using ZnO and Fe_2_O_3_ powders in place of ZFZ-ZnO. GO membranes were prepared using the same GO dispersion quantity as in Ag@ZFZ-GO.

**Water Flux Experiment**

In this experimental setup, the membrane specimen was aligned and secured on a sophisticated filtration unit. Thereafter, an aliquot of 5 mL of deionized water was methodically administered under a defined isotropic pressure. Rigorous quantification ensued, encompassing the temporal interval necessary to attain a specified filtrate volume and the vacuum pressure parameters maintained throughout the suction filtration procedure. Calculate the water flux through the membrane using the following Equation (1)^[2]^:

 (1)

Here, *W_F_* denotes the water flux, quantified in units of L m^-2^ h^-1^ bar^-1^. *V* symbolizes the volume of the introduced deionized water, measured in liters (L). *A* corresponds to the effective contact area of the Vacuum filtration device, expressed in square meters (m²). The *t* (h) signifies the temporal duration of the deionized water's permeation, while *△P* (bar) represents the differential pressure observed during the permeation of the deionized water.

**Ionic Solution Separation Experiment**

A permeate stock solution with a concentration of 5×10^-3^ mol L^-1^, comprising Na^+^, K^+^, L^i+^, and Mg^2+^ ions, was prepared. Different membranes were placed in a separation apparatus, positioned between the permeate solution and an equal volume of deionized water. Samples were periodically collected at specified intervals ranging from 0.17 to 24 h under stirring conditions, for ICP-OES analysis. The data obtained were used to calculate membrane permeability and separation factors according to Equations (2) and (3)^[3, 4]^, respectively.

 (2)

$R=1-P$ (3)

 (4)

In this formula, *P* represents the permeation percentages (Pct. %), *C* denotes the concentration of the test sample, and *C_0_* indicates the original concentration (in mol L^-1^). R represents the ion rejection. *SF* represents separation factor, *P1 >P2*.

The filtration experiments were carried out using a homemade positive pressure device in the laboratory with an effective area of 12.56 cm^2^ at a pressure of 5 bar. Before formal measurements, the membrane samples were pre-pressurized at 6 bar for 30 min until the water flux stabilized. Then, the test was performed at 25 ℃ and 5 bar. The filtrate concentration was measured by ICP-OES and finally the salt rejection (R, %) of the membranes was determined using Equations (5)^[5, 6]^.

$R=\left(1-\frac{Cp}{Cf}\right)\times100\%$ (5)

where *Cp* and *C_f_* are the concentrations of permeate and feed solutions, respectively.

**Ion transport energy barriers measurement**

Measurements were carried out in a thermostatic water bath with magnetic stirring set at 5, 15, 25, 35 and 45°C. The energy barriers (Ea) for ions to cross the membrane can be calculated using an Arrhenius-type Equation(6)^[7-9]^.

$\ln(P)=\ln(\alpha)-\left(\frac{E_{a}}{R}\cdot\frac{1}{T}\right)$ (6)

where *α* is the exponential prefactor, *R* (1.985 × 10^-3^ kcal mol^-1^ K^-1^) is the gas constant, *T* (K) is the temperature, and *Ea* (kcal mol^-1^) is the energy barrier. An Arrhenius plot of the natural logarithm of the ion permeation rate (*P*, (mol m^-2^ h^-1^)) was created at each reciprocal of the absolute temperature. Then, the determination of the slope of the Arrhenius plot, which is related to the energy barrier divided by the gas constant *R*.

**Simulated Salt Lake Separation Experiment**

The composition of the simulated salt lakes mirrored that of Jie Ze Chaka Salt Lake, Tibet, China, with Li^+^ concentration at 0.19 g/L, K^+^ at 2.61 g/L, Na^+^ at 44.24 g/L, and Mg^2+^ at 0.36 g/L. The separation process followed typical protocols for ionic solutions. *P* and *SF* were determined according to Eqs. (2) and (4), respectively.

**Real Salt Lake Separation**

Separation experiments in real complex systems utilized Jieze Chaka Salt Lake. The procedure paralleled typical ionic solution separation methods, adjusting the pH with HCl and NH₃·H₂O for experiments at pH=2, pH=4, and pH~7 (using deionized water). *P* and *SF* were determined according to Eqs. (2) and (4), respectively.

This location is renowned for its rich mineral content and complex ionic composition, making it an ideal site for studying selective ion separation. The water samples were collected from the surface layer (0-50 cm) of the salt lake under specific time and environmental conditions to ensure their representativeness. Immediately upon collection, the samples were stored in pre-cleaned high-density polyethylene (HDPE) bottles and transported to the laboratory under refrigerated conditions at 4°C to prevent any alteration in composition. Prior to experimentation, the salt lake water samples underwent precipitation to remove suspended and particulate matter, ensuring the experimental process was free from interference by impurities. The concentrations of major ions such as Na⁺, K⁺, Li⁺, Mg²⁺, among others, were determined using inductively coupled plasma optical emission spectroscopy (ICP-OES) to verify the ionic compositions and concentrations, thus ensuring consistency with the actual salt lake water environment.

# Theoretic calculation

**Density functional theory**

Spin-polarized first-principles calculations were conducted using the Vienna Ab initio Simulation Package (VASP)^[10]^. The projection-enhanced wave (PAW) method described ion-electron interactions, while Perdew-Burke-Ernzerhof (PBE) approach were applied to electron exchange-correlation energy interactions^[11-13]^. The Monkhorst-Pack k-point grid was set at 6 × 6 × 1. Van der Waals interactions were addressed using the Grimme scheme (DFT-D3)^[14, 15]^. The Plane-wave Cutoff Energy was set at 600 eV, with energy and force convergence thresholds of 10^-5^ eV and 1 × 10^-3^ eV Å^-1^, respectively. The adsorption energies (*E_ads_*) were defined as^[16]^:

 (5)

where *E_*A_*, *E_A_*, and *E_*_* are the total energies of adsorbed system.

**Molecular dynamics simulations** This study employed classical molecular dynamics (MD) simulations to elucidate the mechanism of ZnFe_2_O_4_-ZnO formation. NVT (constant particle number, volume, and temperature) system synthesis simulations were conducted using VASP for structural state calculations^[17]^. The Nosé-Hoover thermostat method maintained the temperature at 773.15 K throughout the simulations. Integration of the equations of motion was performed using the Stoermer-Verlet time integration algorithm with a time step of 1 fs and a total of 137,240 molecular dynamics steps^[17]^.

# Figures


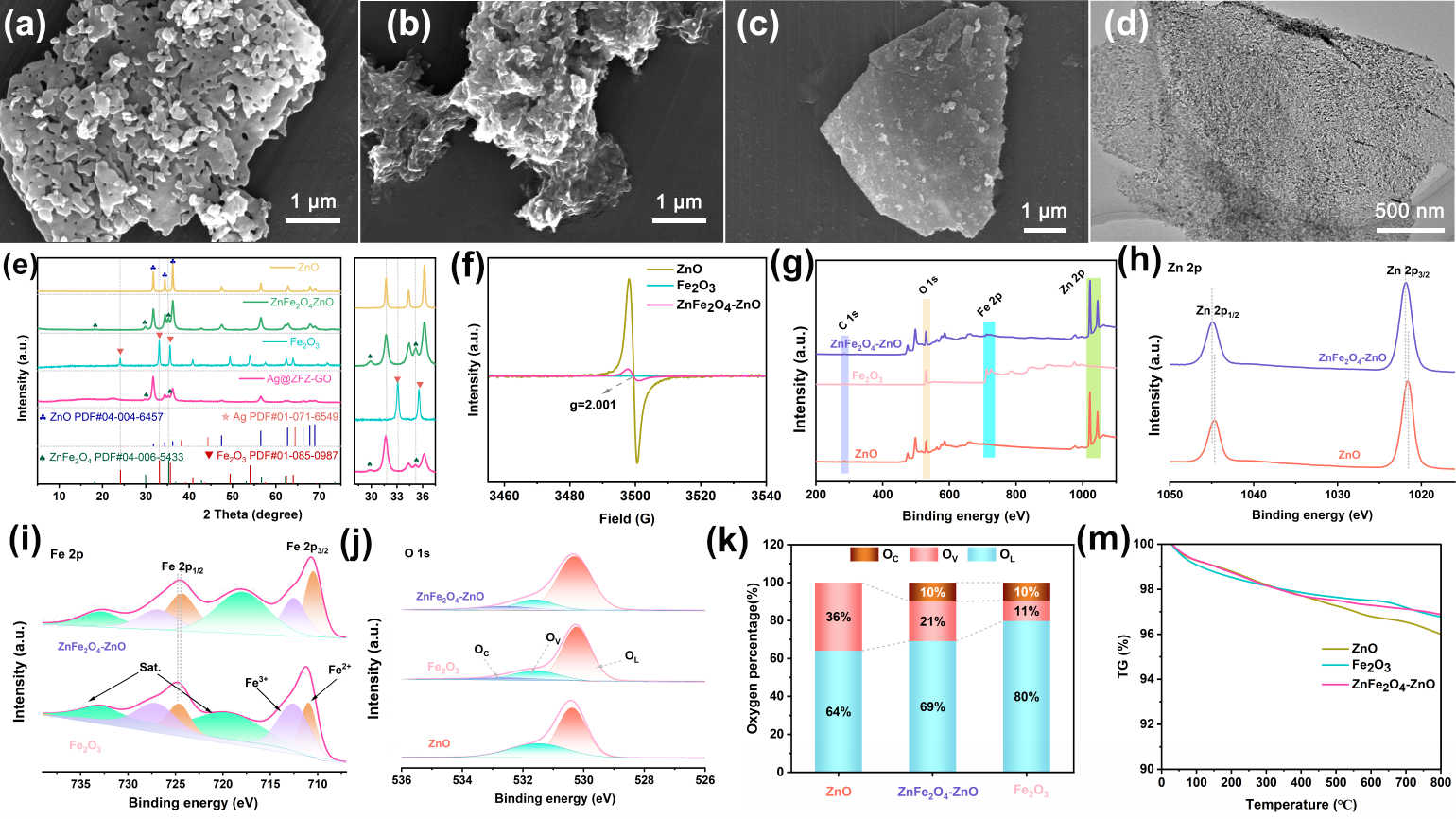


**Fig. S1.** Structural characterization of ZnFe_2_O_4_-ZnO porous nanosheets, ZnO, Fe_2_O_3_. The SEM (a, b, c) and (d) TEM images of prepared ZnO, Fe_2_O_3_, and ZnFe_2_O_4_-ZnO. (e) XRD patterns and amplified XRD patterns of selected areas; (f) EPR spectra; (g) XPS spectra for survey; (h) Zn 2p; (i) Fe 2p; (j) O1s; (k) Percentage of each oxygen species; and (m) TGA patterns.


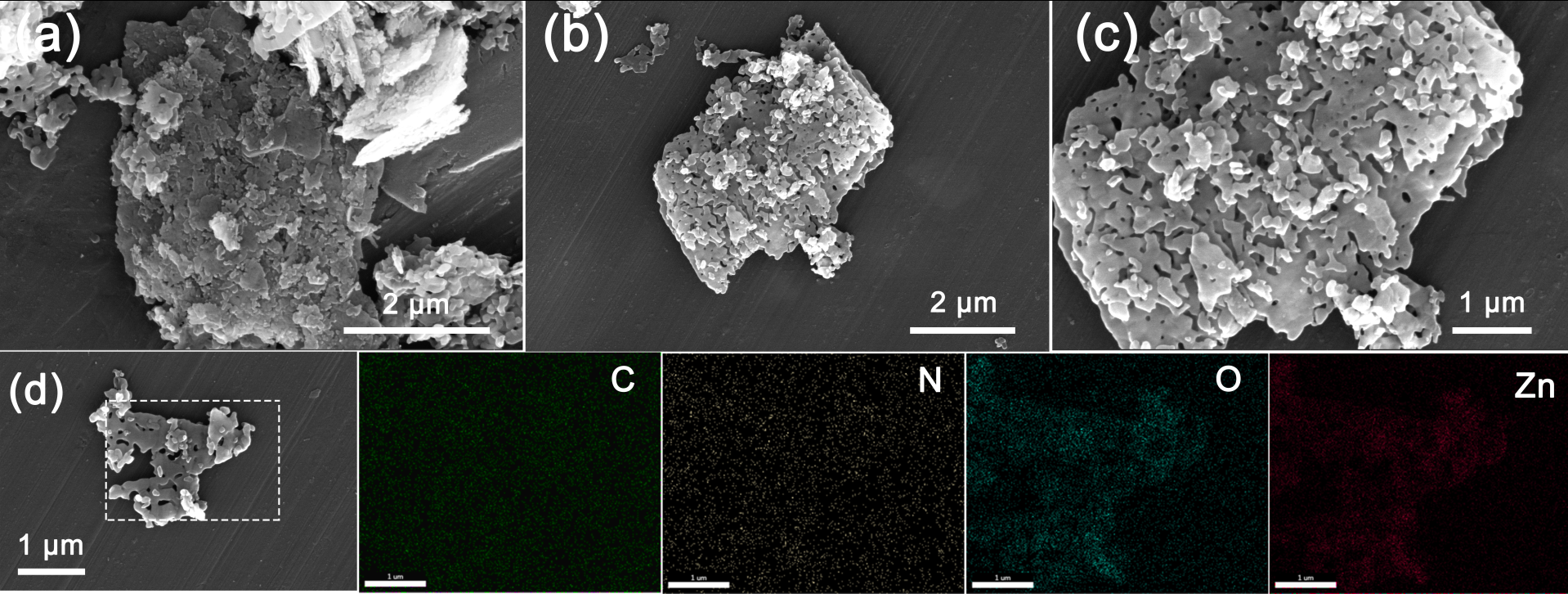


**Fig. S2.** SEM images (a, b, c) and Elemental mapping images (d) of prepared ZnO.


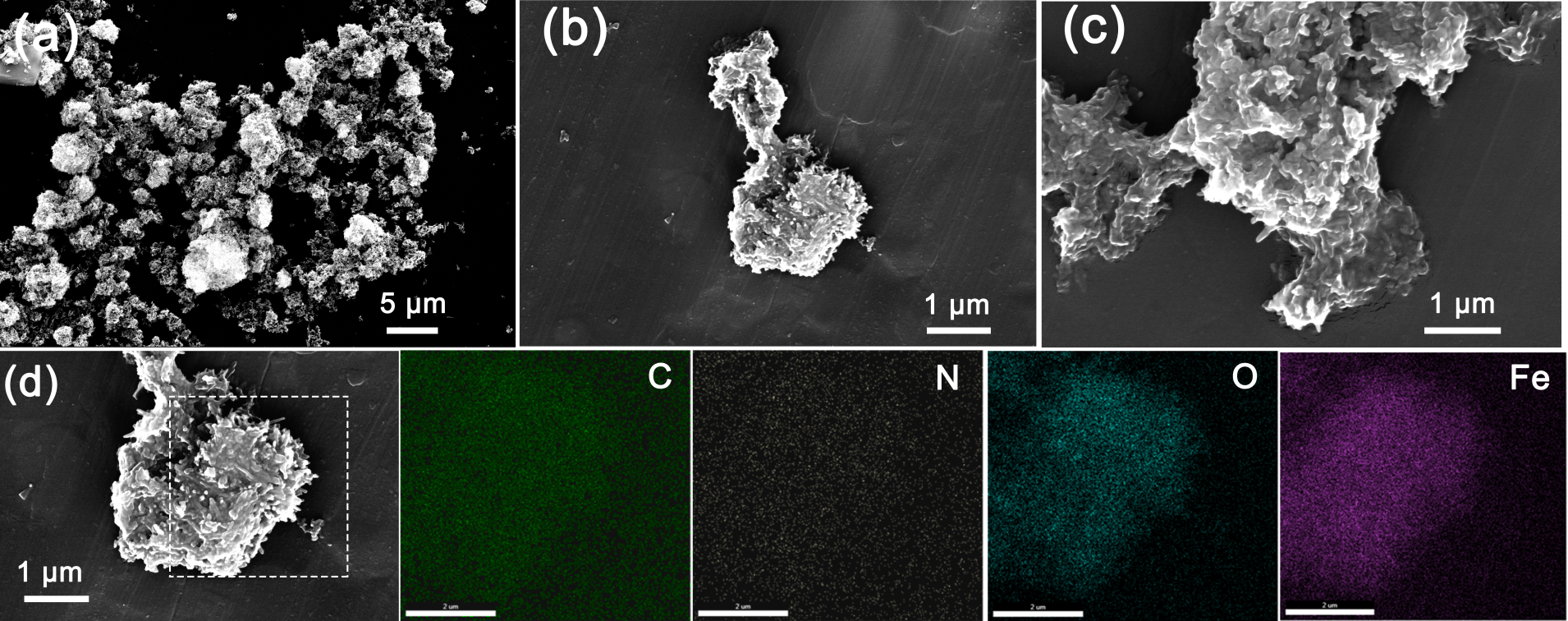


**Fig. S3.** SEM images (a, b, c) and Elemental mapping images (d) of prepared Fe_2_O_3_.


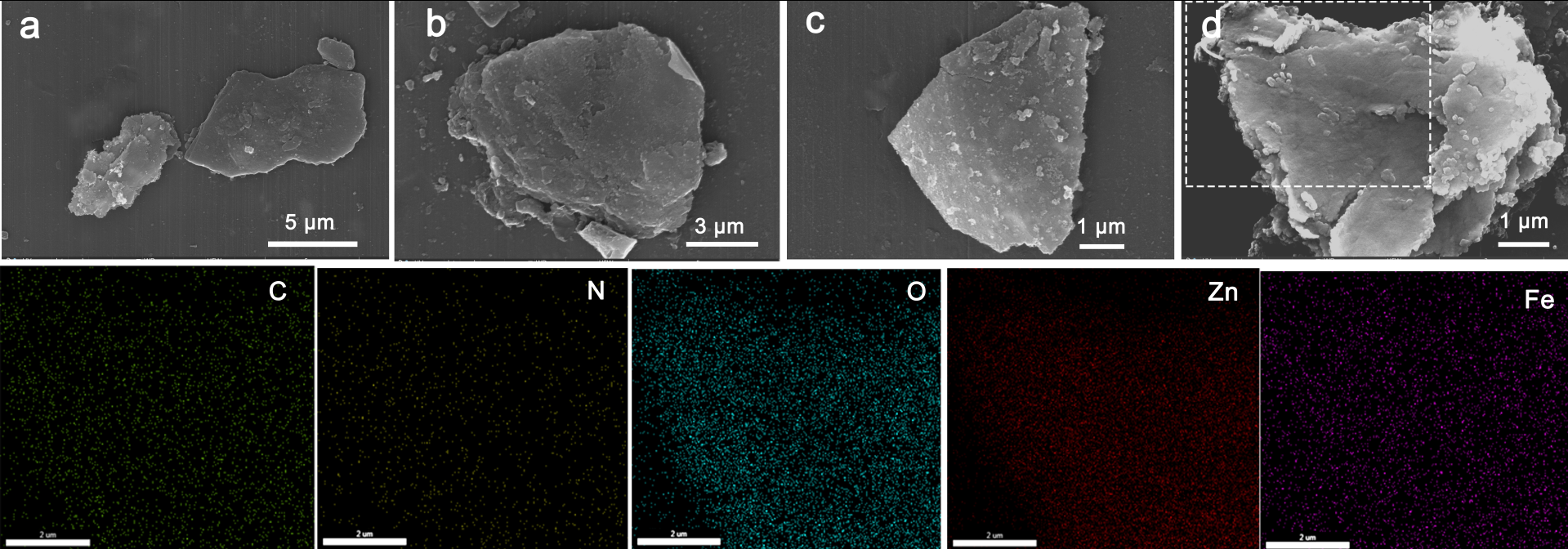


**Fig. S4.** SEM images (a, b, c) and Elemental mapping images (d) of prepared ZnFe_2_O_4_-ZnO.


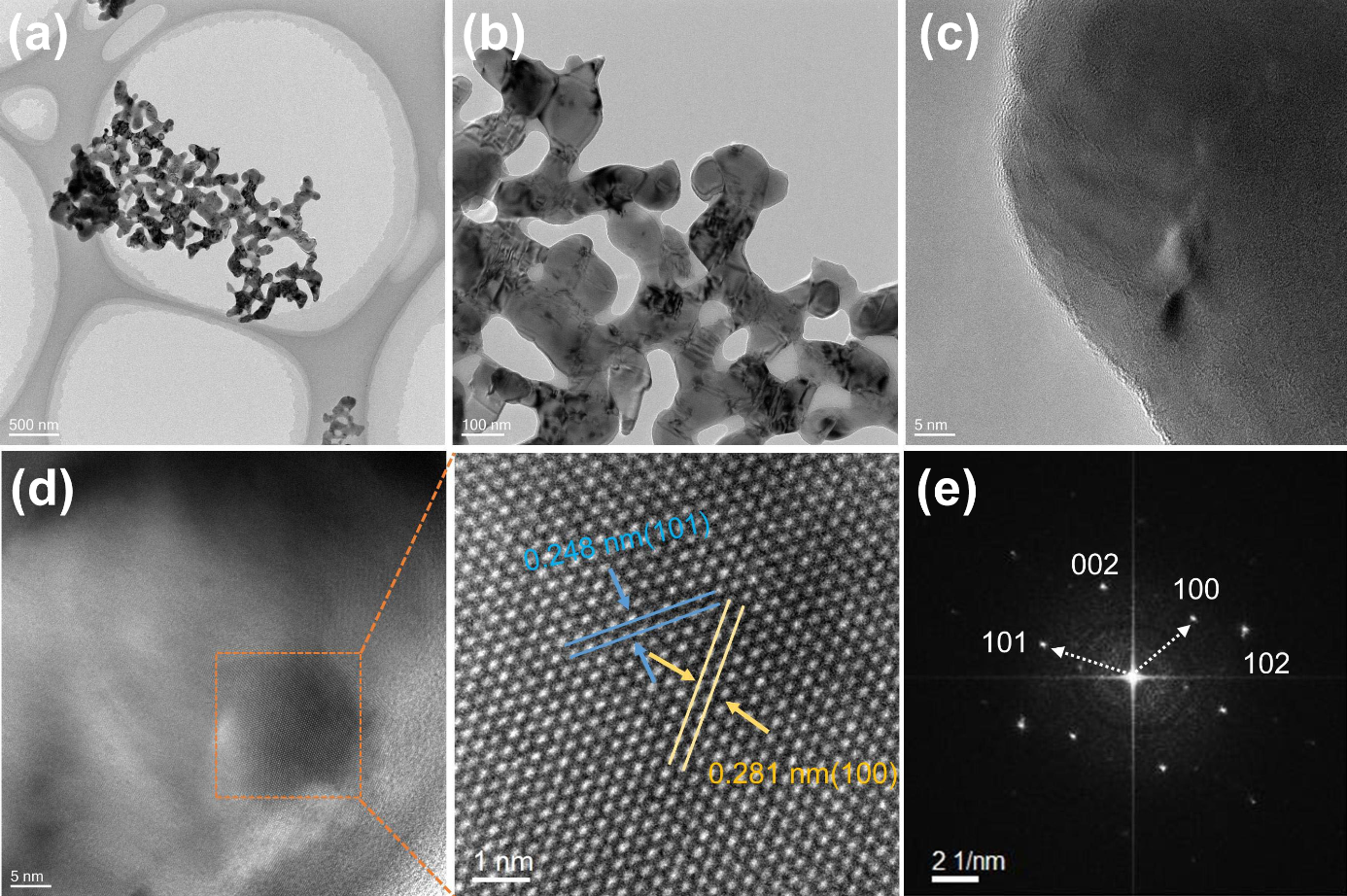


**Fig. S5.** The TEM (a, b, c), HR-TEM(d) and its enlarged images and corresponding FFT patterns (e) of the prepared ZnO.


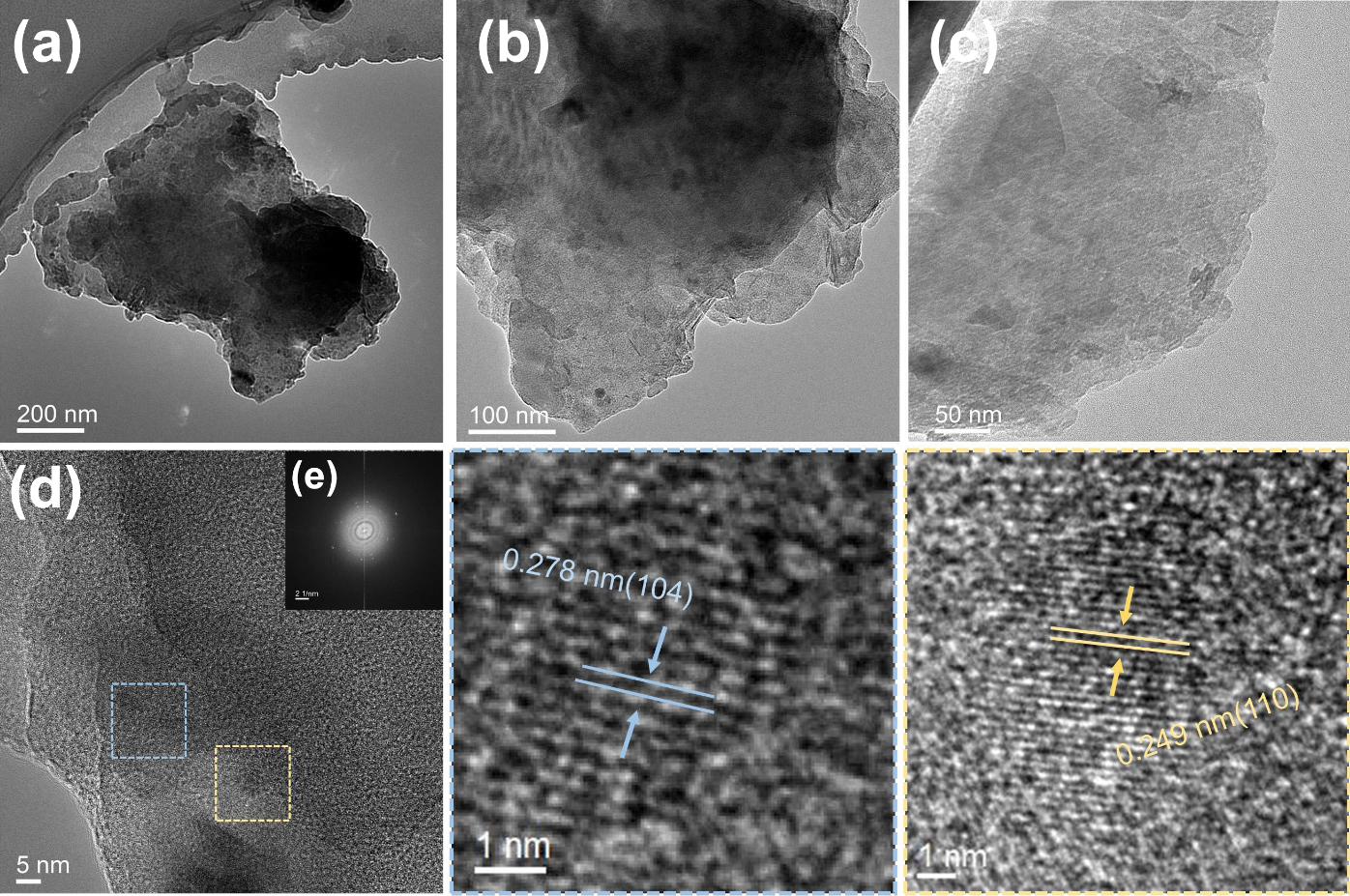


**Fig. S6.** The TEM (a, b, c), HR-TEM(d) and its enlarged images and corresponding FFT patterns of the prepared Fe_2_O_3_.


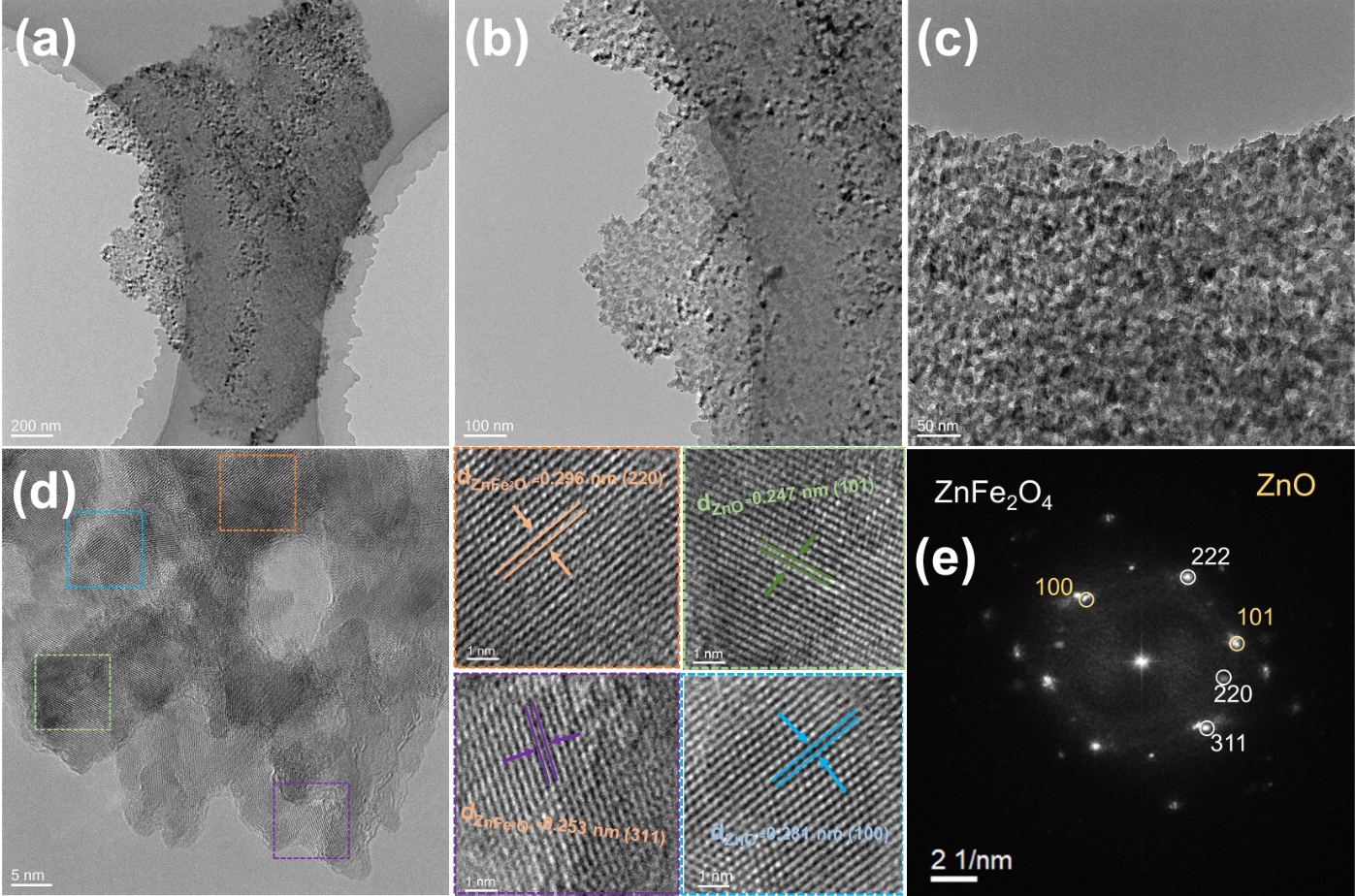


**Fig. S7.** The TEM (a, b, c), HR-TEM(d) and its enlarged images and corresponding FFT patterns of the prepared ZnFe_2_O_4_-ZnO.


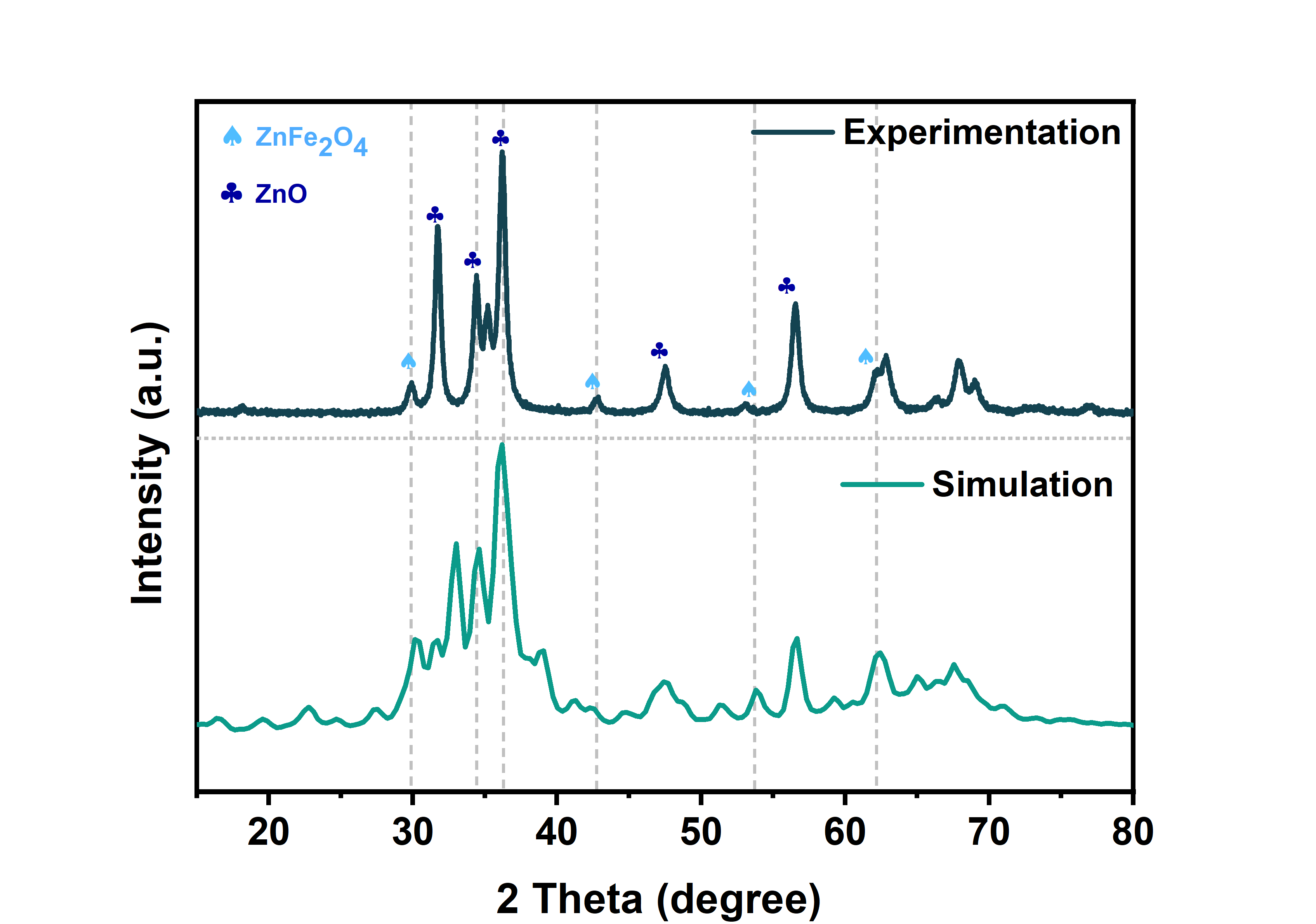


**Fig. S8.** Comparison of experimental and simulated XRD patterns.


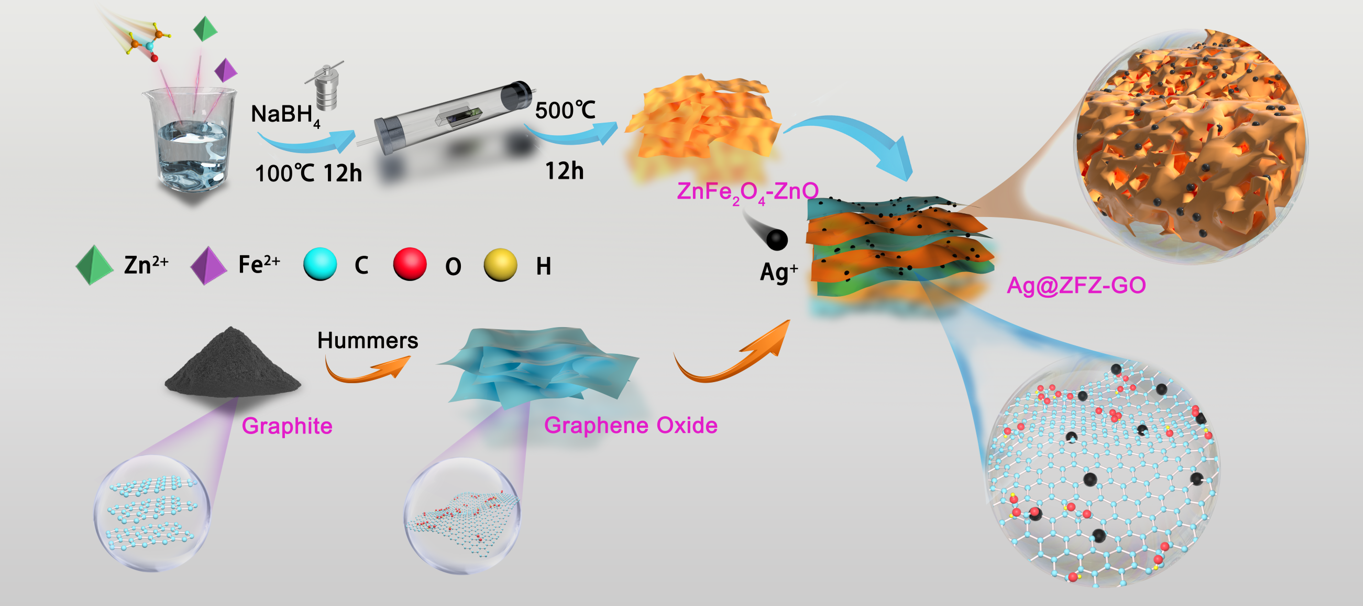


**Fig. S9** Schematic illustration of the synthesis of Ag@ZFZ-GO membrane.


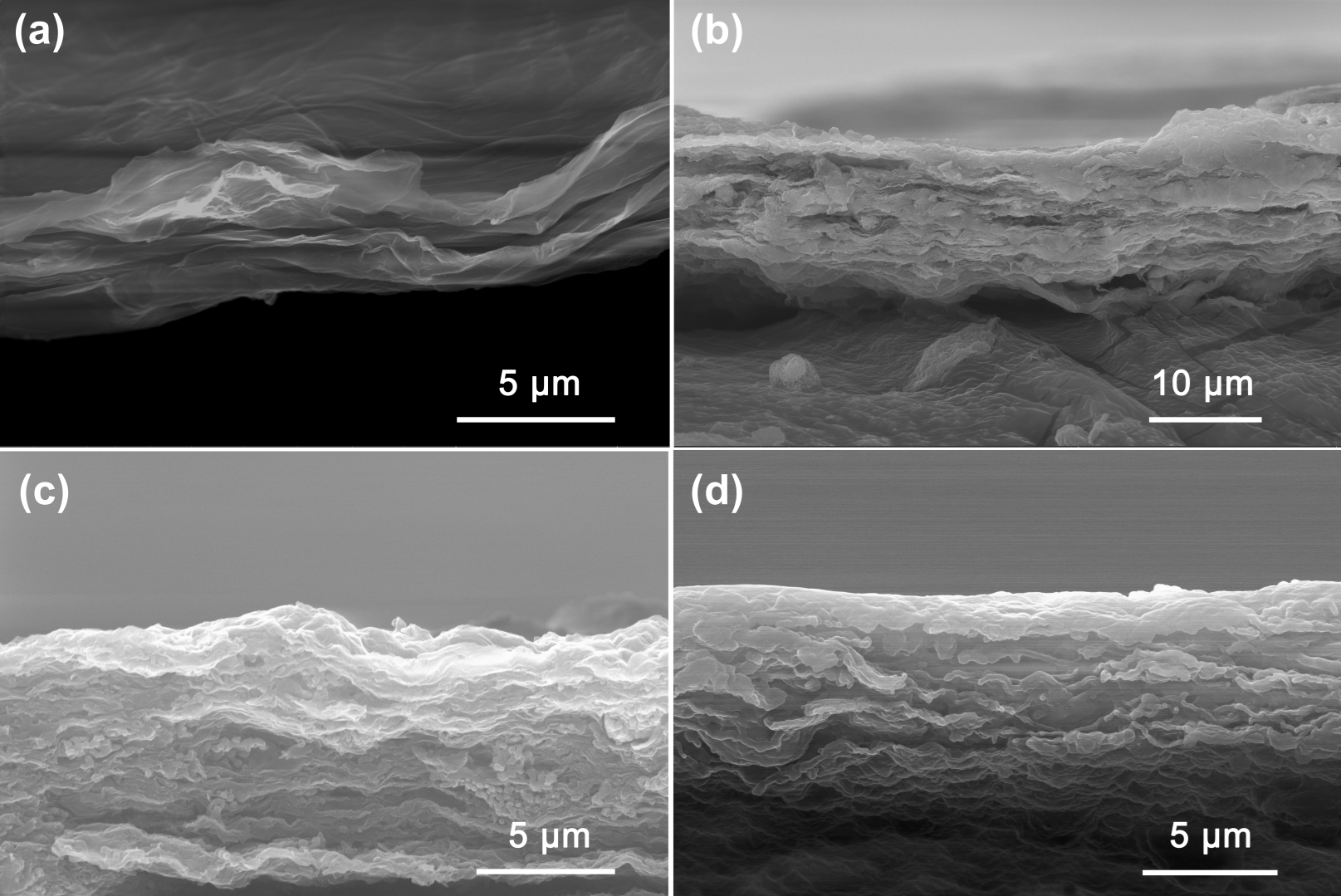


**Fig. S10.** SEM images of GO (a), Ag@ZFZ-GO (b), Ag@ZnO-GO (c), and Ag@Fe_2_O_3_-GO (d) membranes.


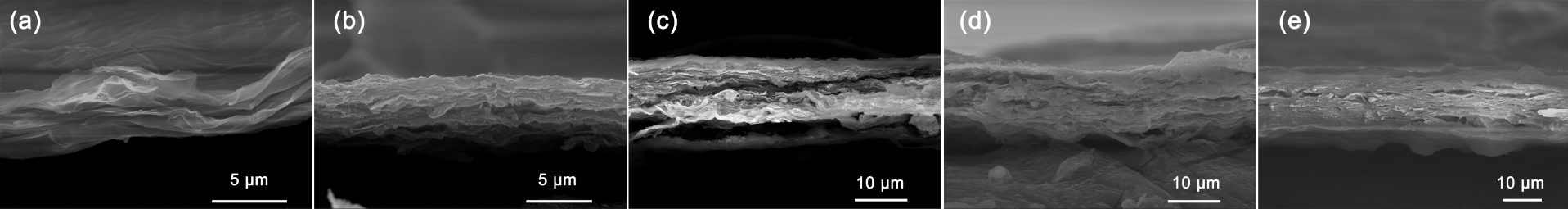


**Fig. S11.** SEM images of GO (a), ZFZ-GO (b), Ag@ZFZ-GO-1 (c), Ag@ZFZ-GO-2 (d), and Ag@ZFZ-GO-3 (e) membranes.


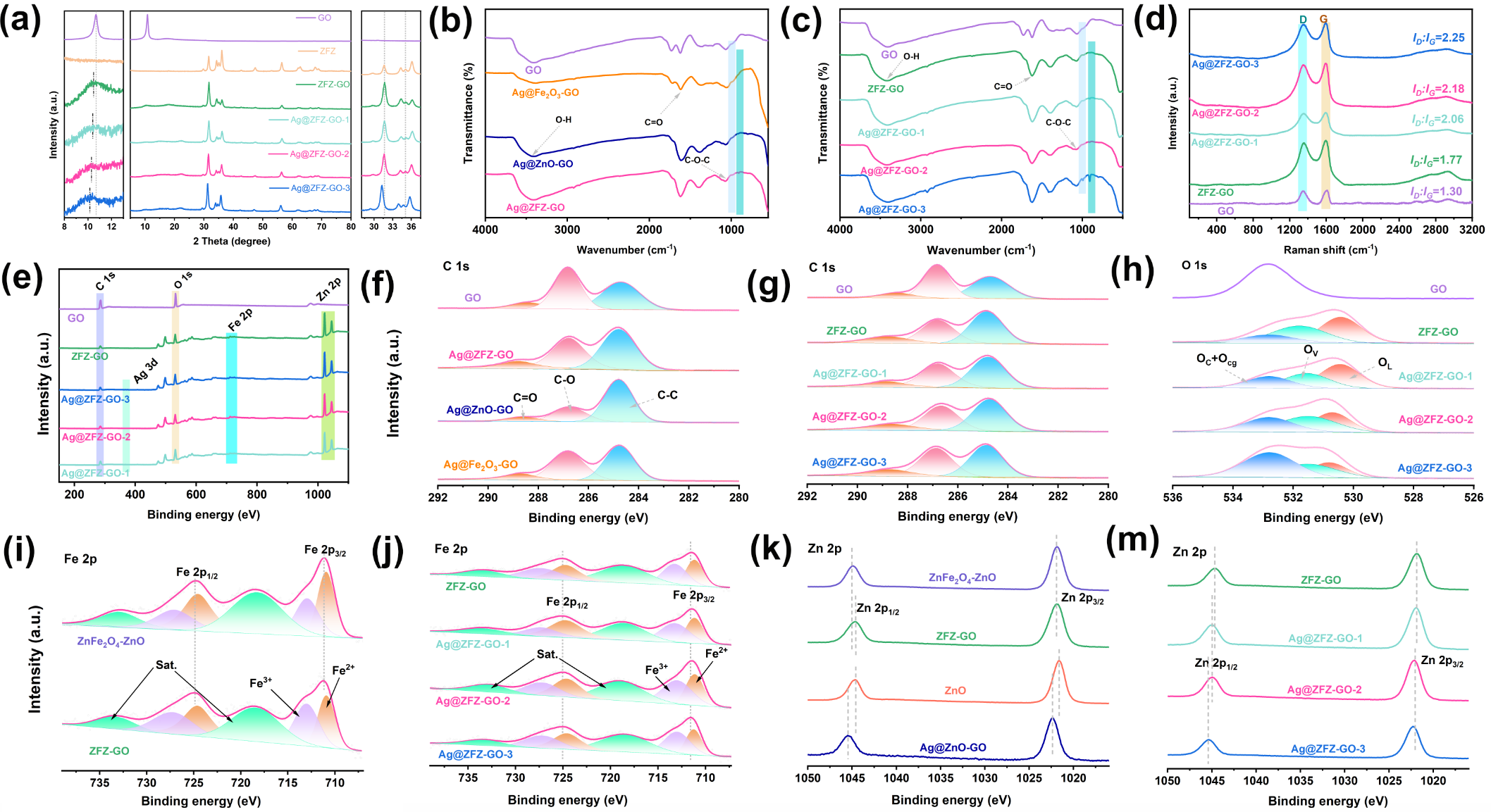


**Fig. S12.** Structural characterization of GO, Ag@ZnO-GO, Ag@Fe_2_O_3_-GO and Ag@ZFZ-GO series membrane materials.; XRD patterns and amplified XRD patterns of selected areas (a); FT-IR patterns (b, c); Raman spectrum (d); XPS spectra for survey (e); C 1s (f, g); O1s (h); Fe 2p (I, j); Zn 2p (k, m).


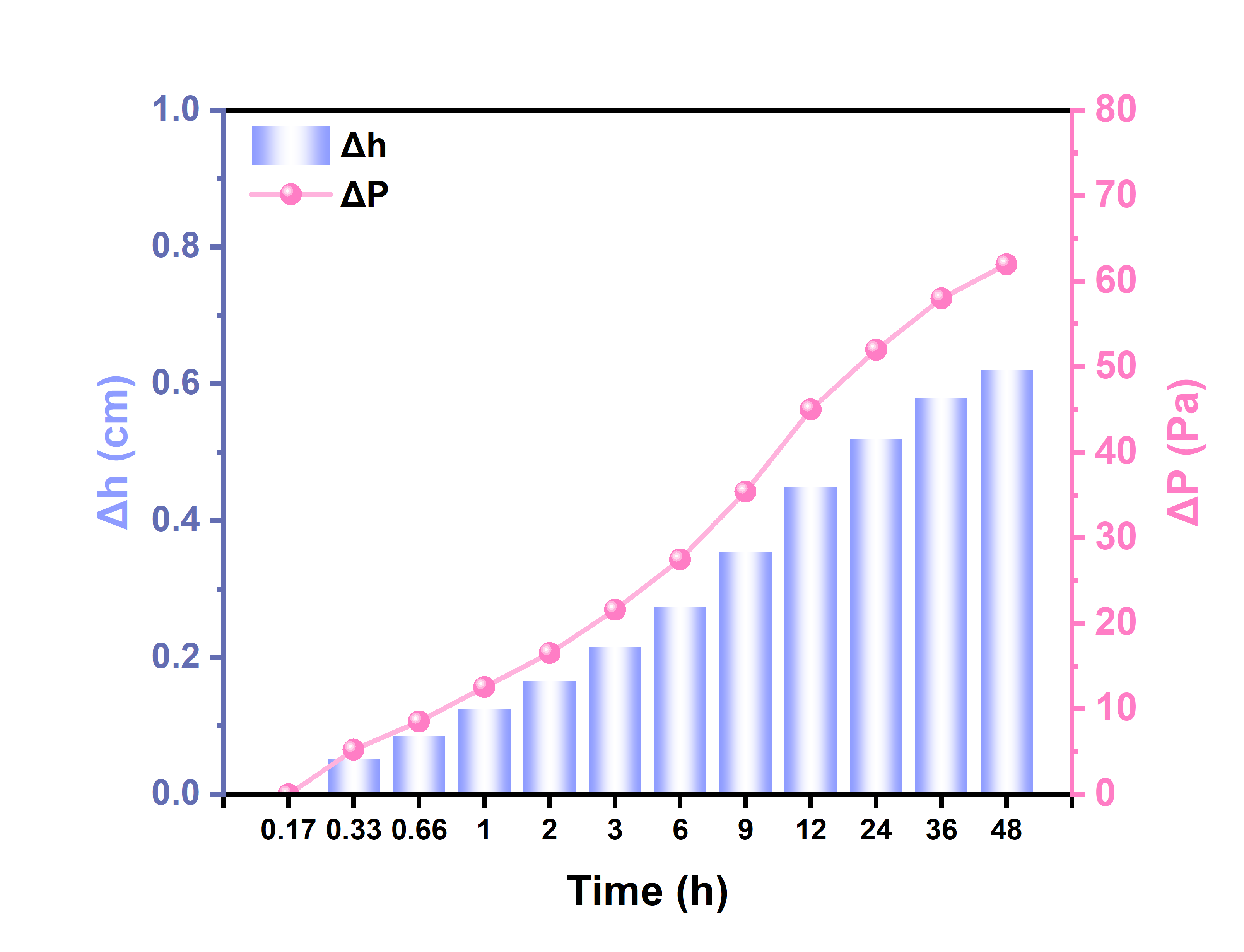


**Fig. S13.** Evolution of the water level difference Δh, and corresponding hydrostatic pressure difference ΔP, between the feed and the permeate during permeation test. ΔP=ρgΔh. Since both the solutions are dilute, ρ takes the value of water density of 1000 kg m^-3^**.**


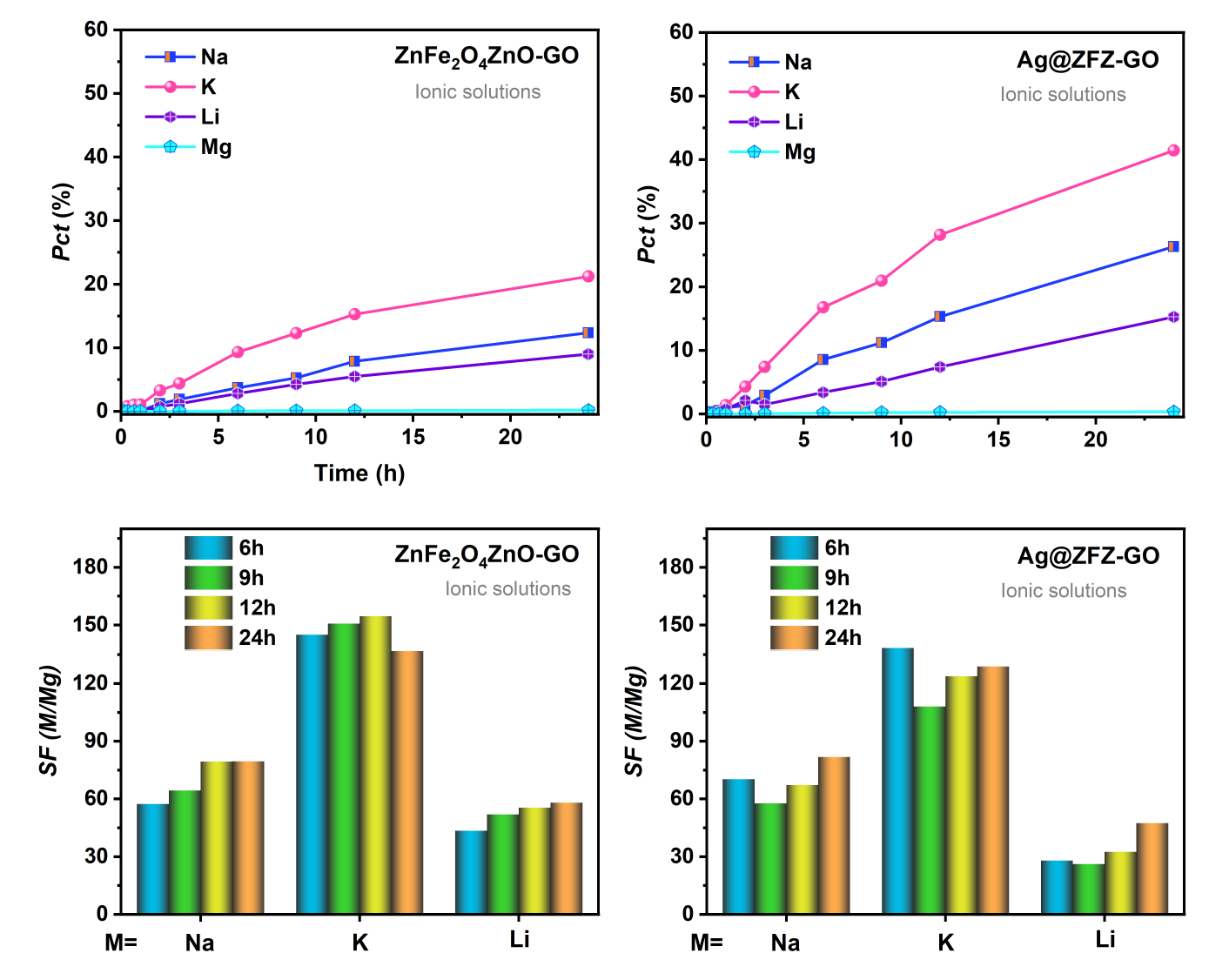


**Fig. S14.** The permeation percentages (Pct.) of ionic solutions as a function of time and the corresponding separation factor of ZFZ-GO membrane and Ag@ZFZ-GO membrane at 6-24h.


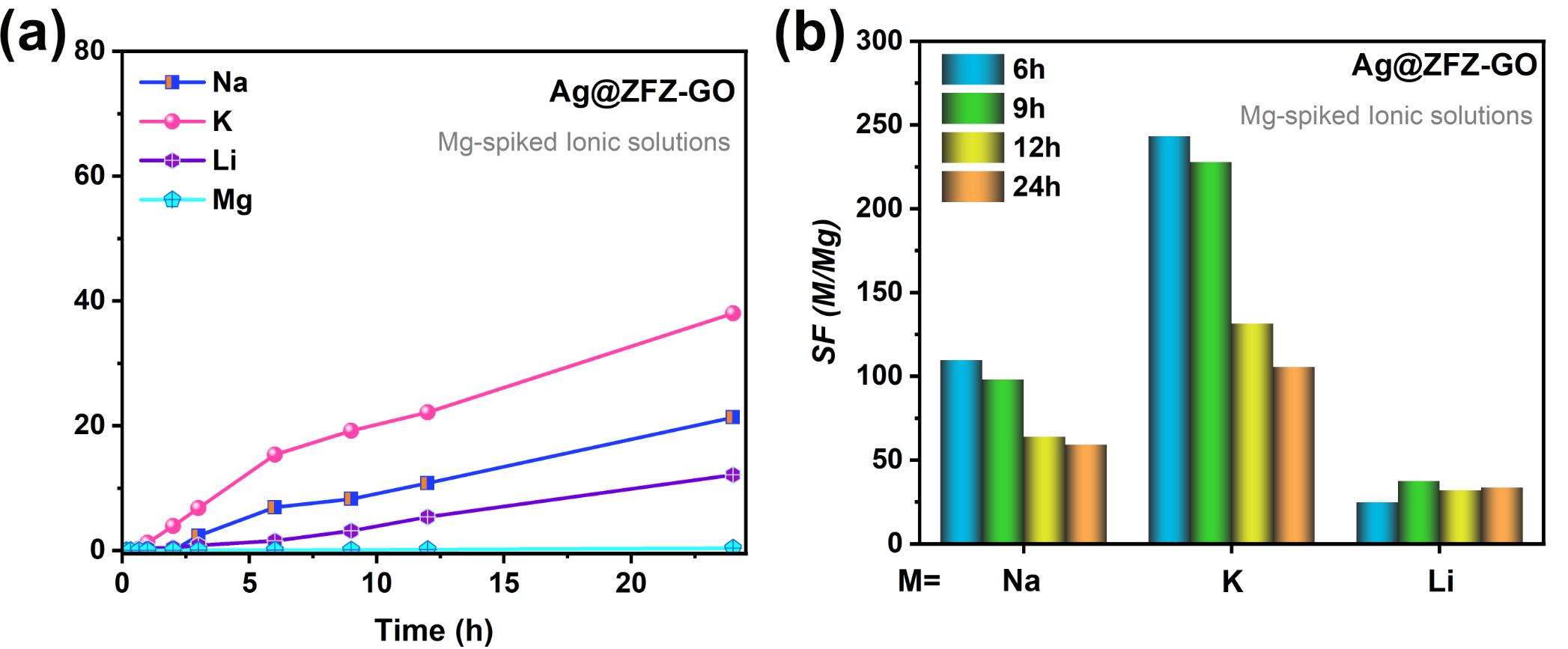


**Fig. S15.** The permeation percentages (Pct.) of ionic solutions as a function of time and the corresponding separation factor of ZFZ-GO membrane and Ag@ZFZ-GO membrane at 6-24h.


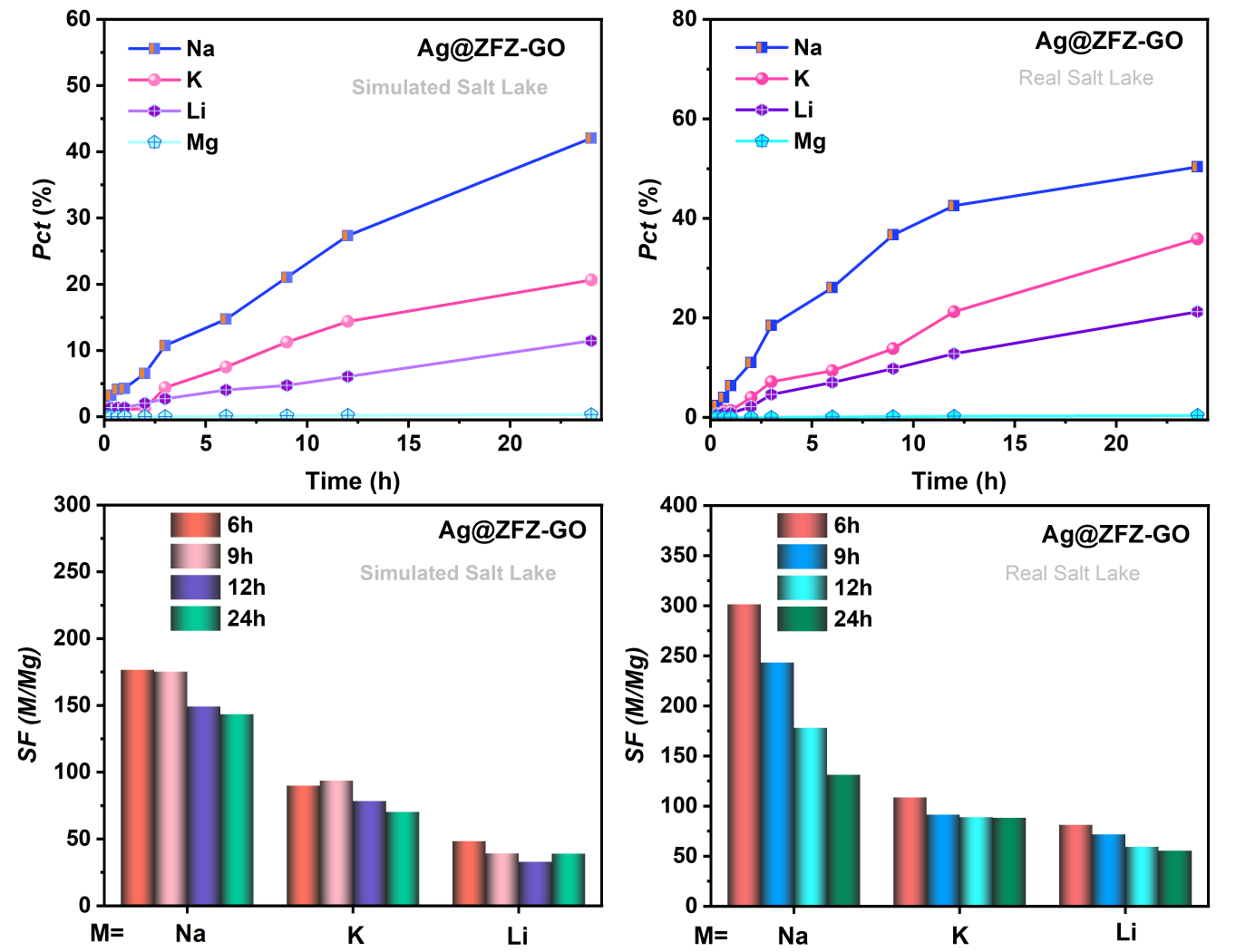


**Fig. S16.** The permeation percentages (Pct.) of simulated salt lakes and real salt lakes as a function of time and the corresponding separation factor of Ag@ZFZ-GO membrane at 6-24h.


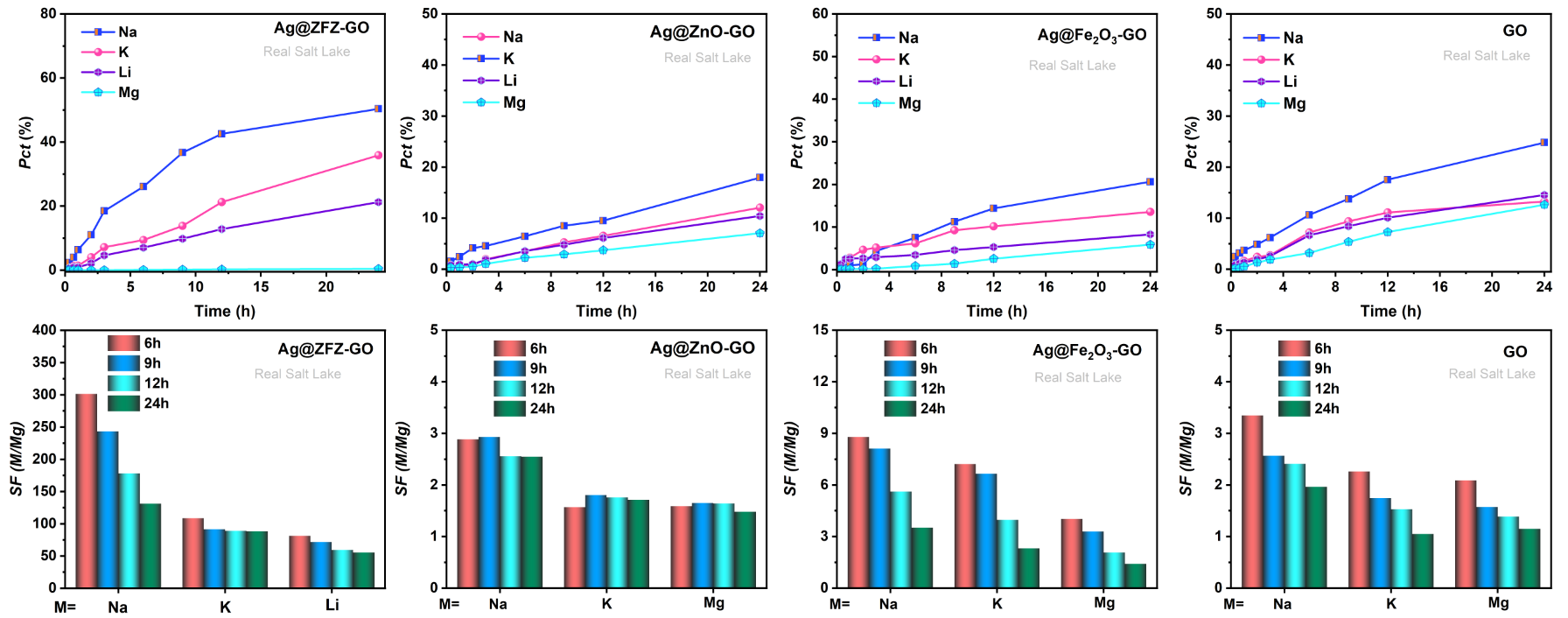


**Fig. S17.** The permeation percentages (Pct.) of real salt lakes as a function of time and the corresponding separation factor of Ag@ZFZ-GO, Ag@ZnO-GO, Ag@Fe_2_O_3_-GO, GO membranes at 6-24h.


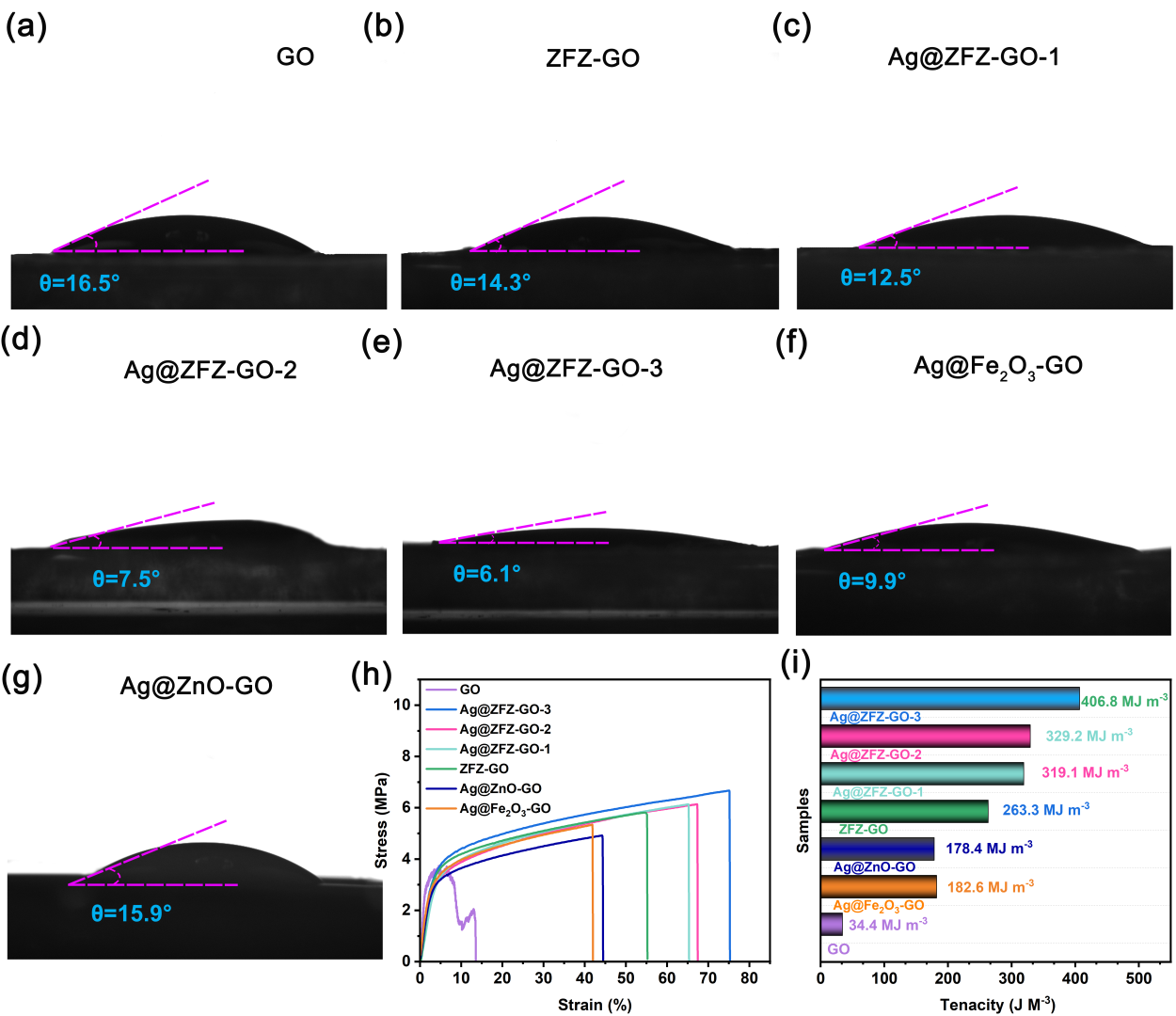


**Fig. S18.** (a-g). The contact angle measurements; (h) typical tensile stress-strain curves; (i) corresponding tenacity values of GO, Ag@ZnO-GO, Ag@Fe_2_O_3_-GO and Ag@ZFZ-GO series membrane materials


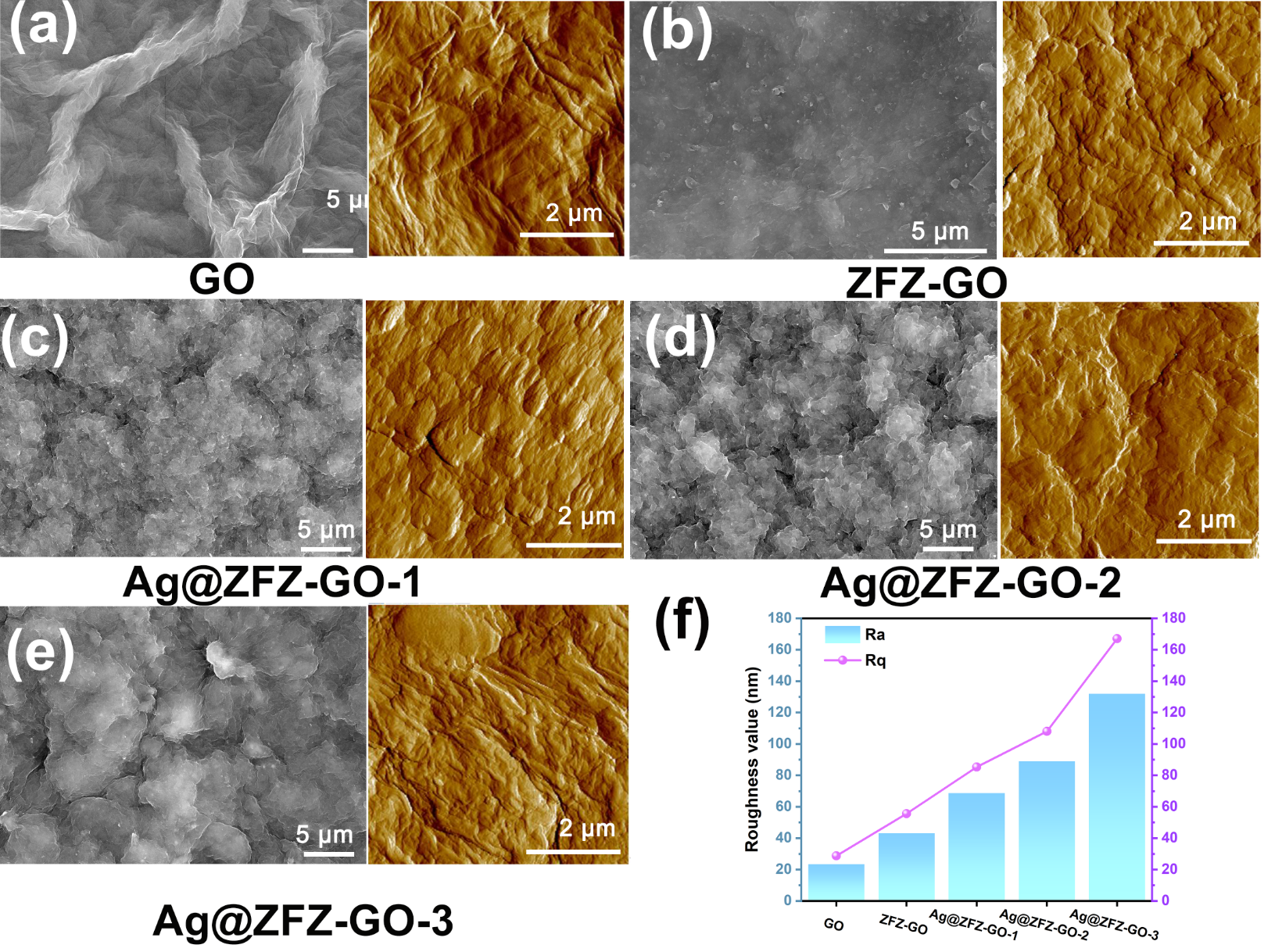


**Fig. S19.** (a-e) SEM surface and AFM topography images of GO, ZFZ-GO and Ag@ZFZ-GO series membranes. (f) Ra and Rq values of different materials.


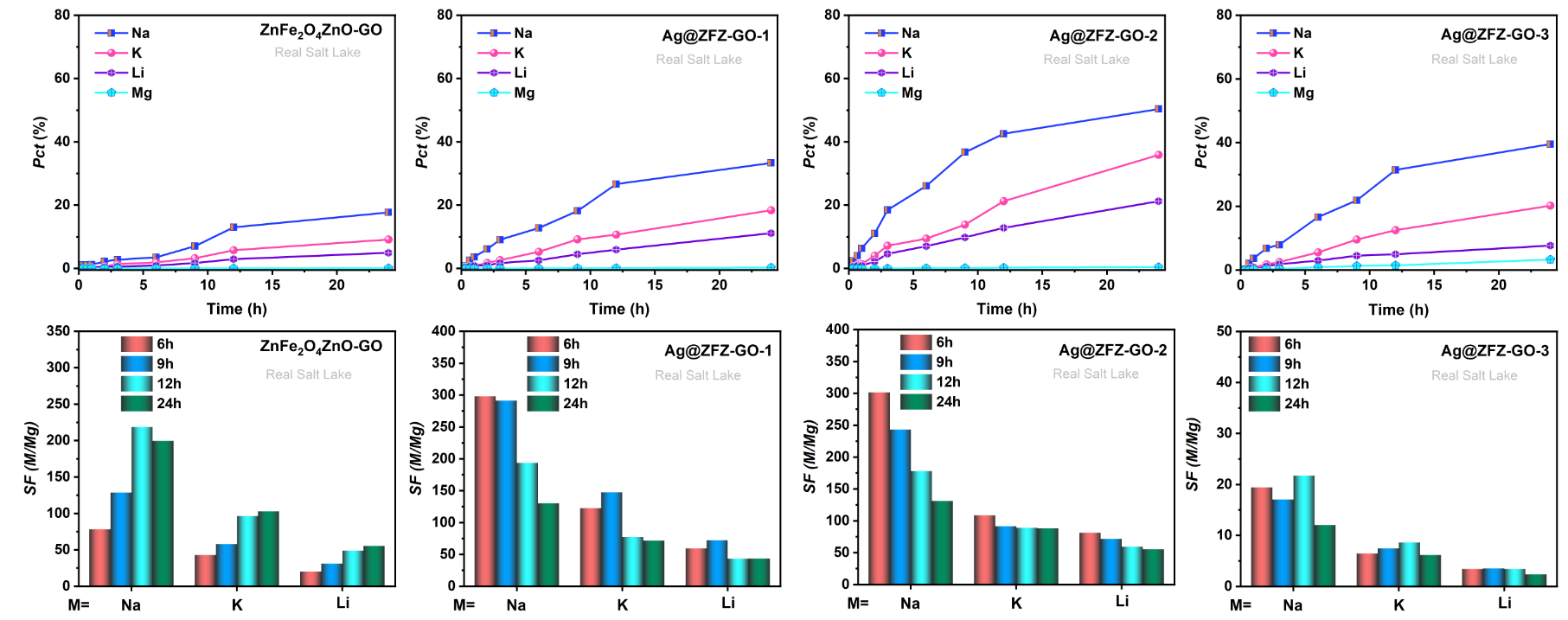


**Fig. S20.** The permeation percentages (Pct.) of real salt lakes as a function of time and the corresponding separation factor of ZFZ-GO membrane, Ag@ZFZ-GO-1, 2, 3 membranes at 6-24h.


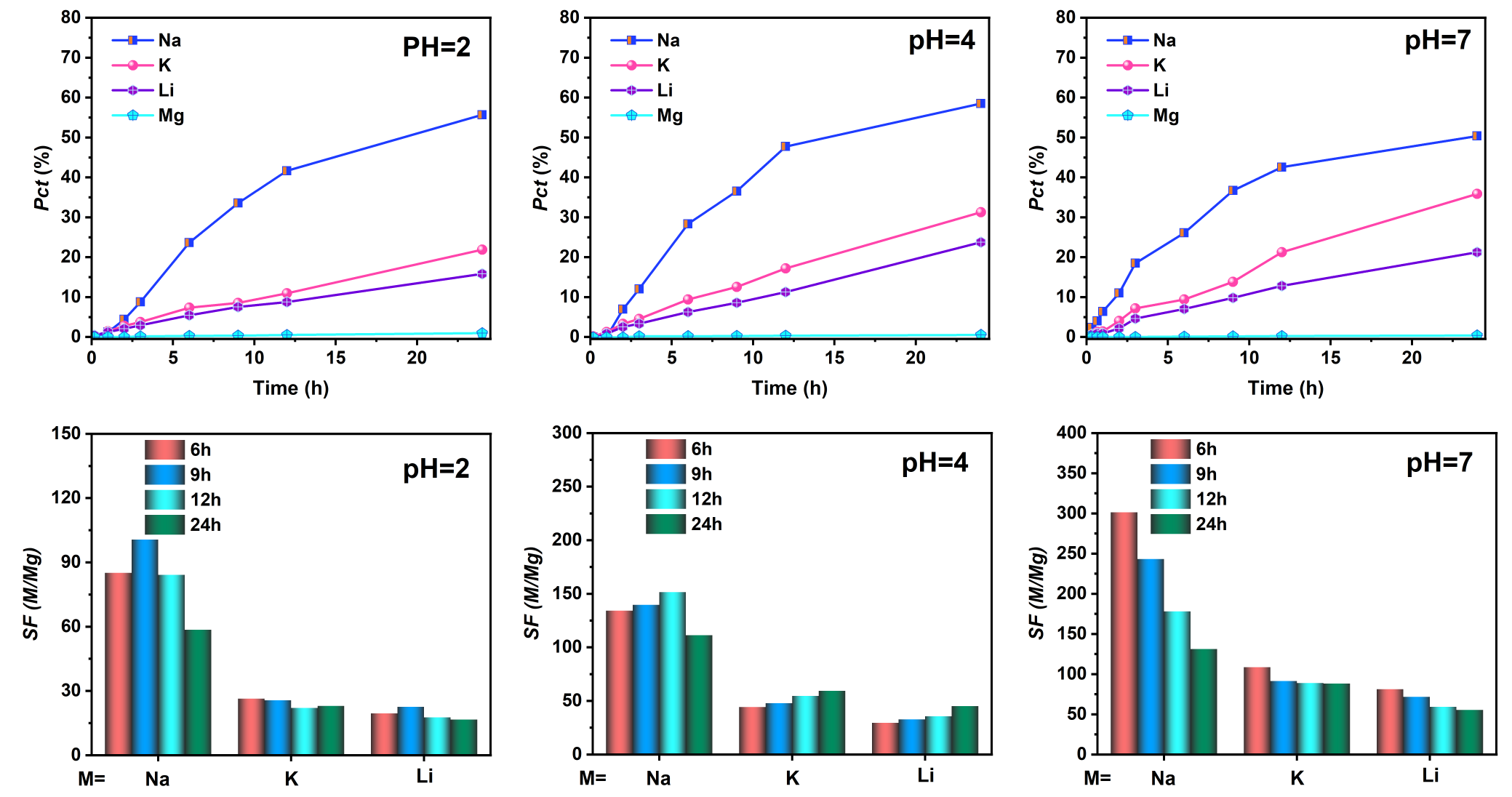


**Fig. S21.** The permeation percentages (Pct.) of real salt lakes at different pH as a function of time and corresponding separation factors for Ag@ZFZ-GO membrane at 6-24h.


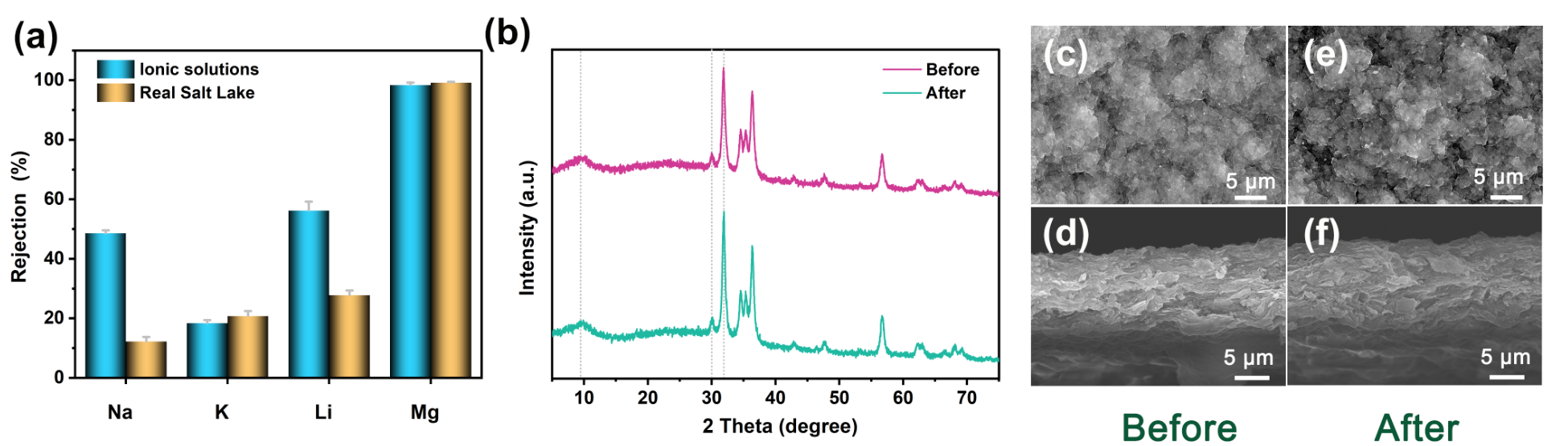


**Fig. S22.** (a) Ion rejection of Ag@ZFZ-GO membrane in equal concentration ionic solution and real salt lake. (b) XRD analysis before and after nanofiltration separation. SEM images before (c-d) and after (e-f) nanofiltration separation.


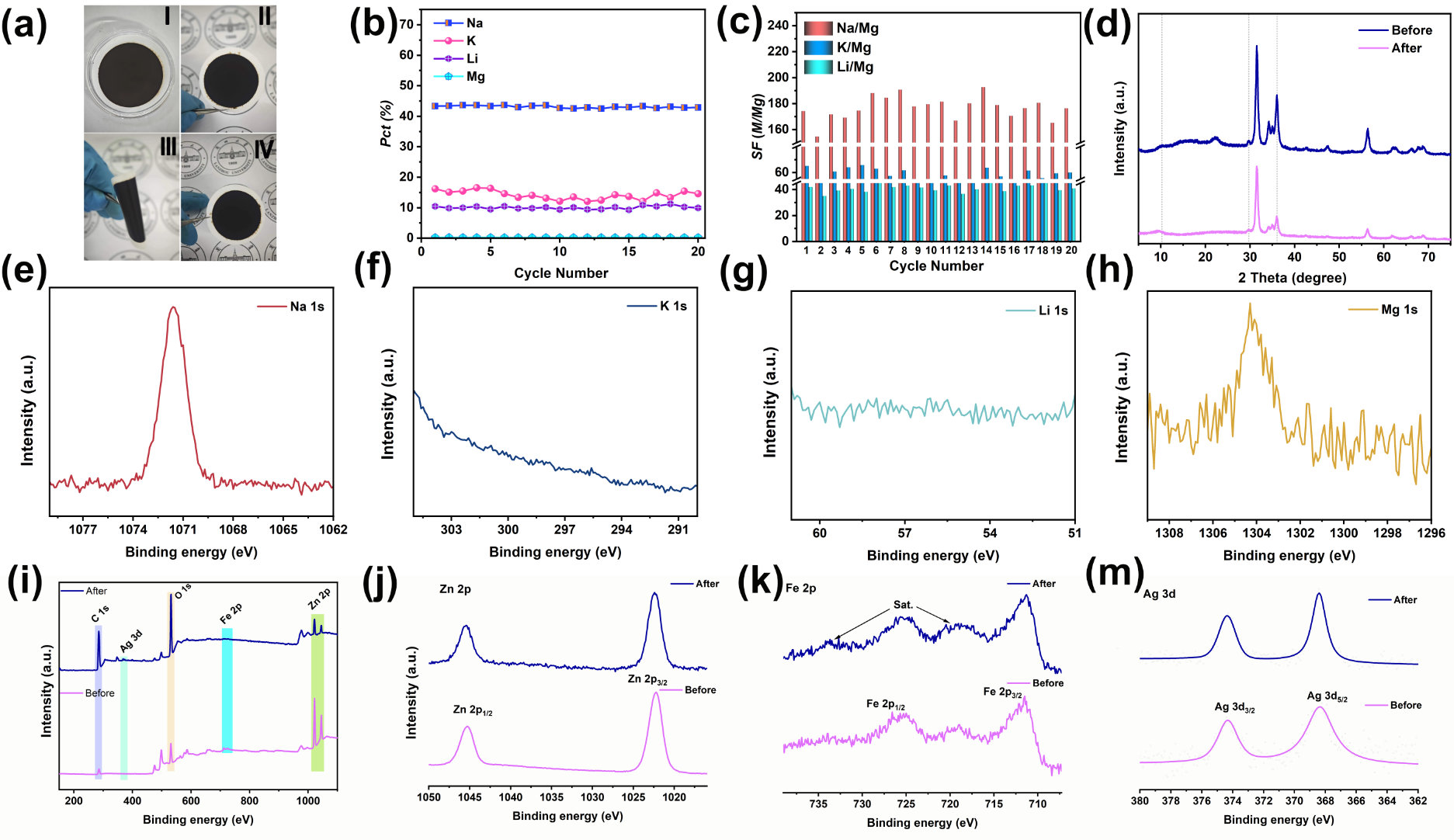


**Fig. S23.** The stability measurement of Ag@ZFZ-GO membranes. (a) The digital images of folding; (b, c) Pct. and corresponding separation factors for 20 cycling tests (12 h each) in real salt lakes; (d) XRD patterns before and after cycling; (e-m) XPS spectra of after cycling.


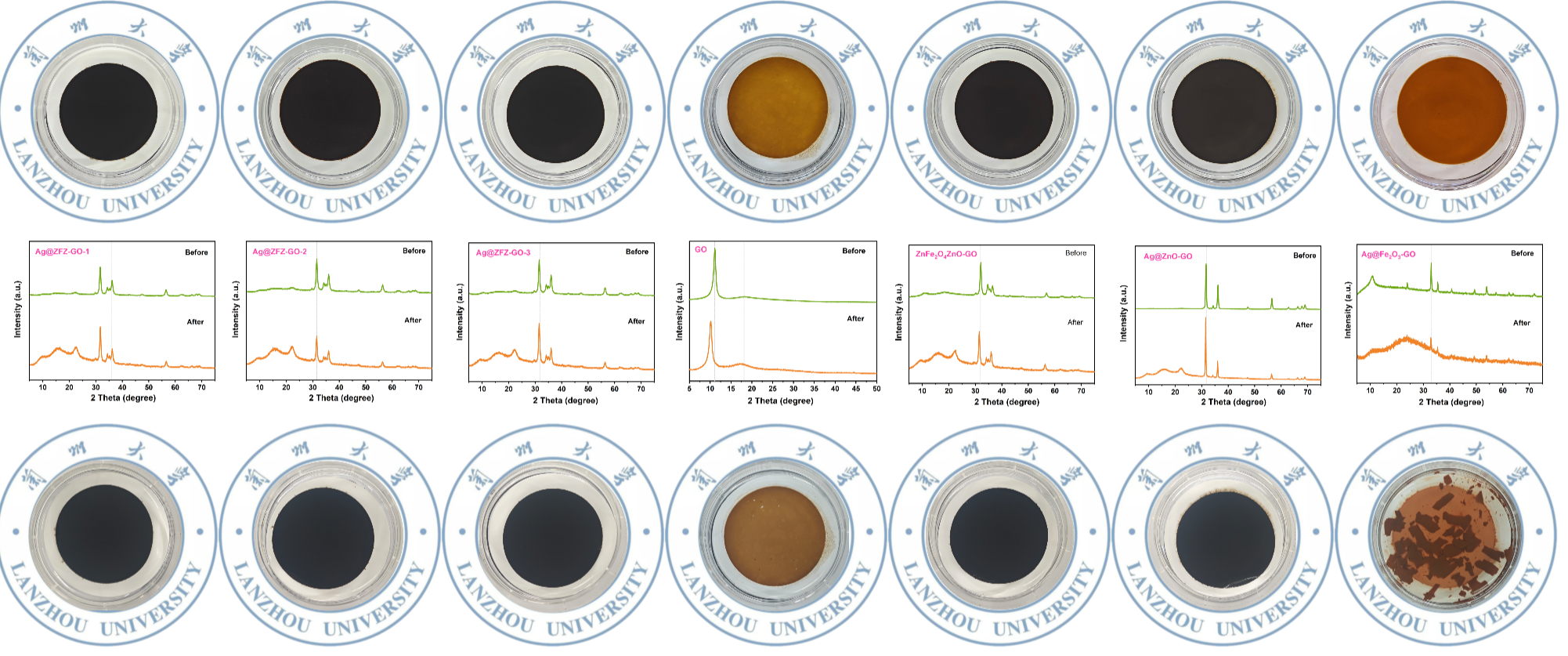


**Fig. S24.** The digital images of GO, Ag@ZnO-GO, Ag@Fe_2_O_3_-GO and Ag@ZFZ-GO series membrane materials before and after immersion in neutral deionized water and corresponding XRD patterns.


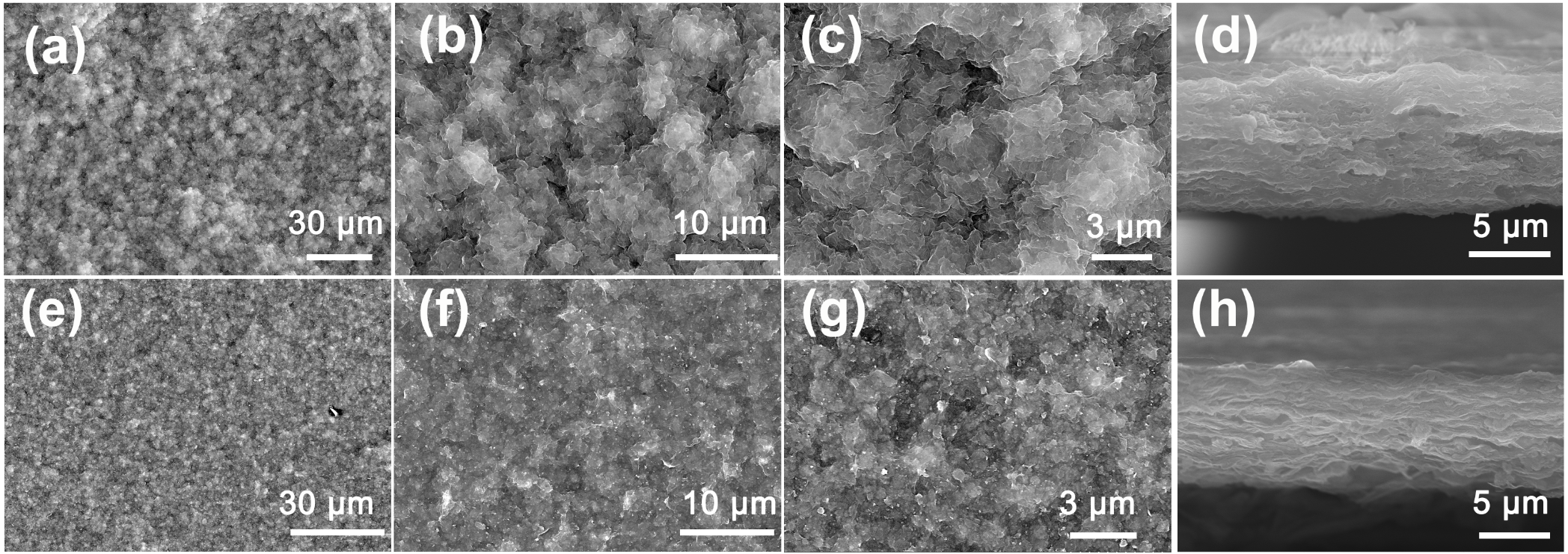


**Fig. S25. The SEM images of** before (a-d) and after (e-h) cycling.


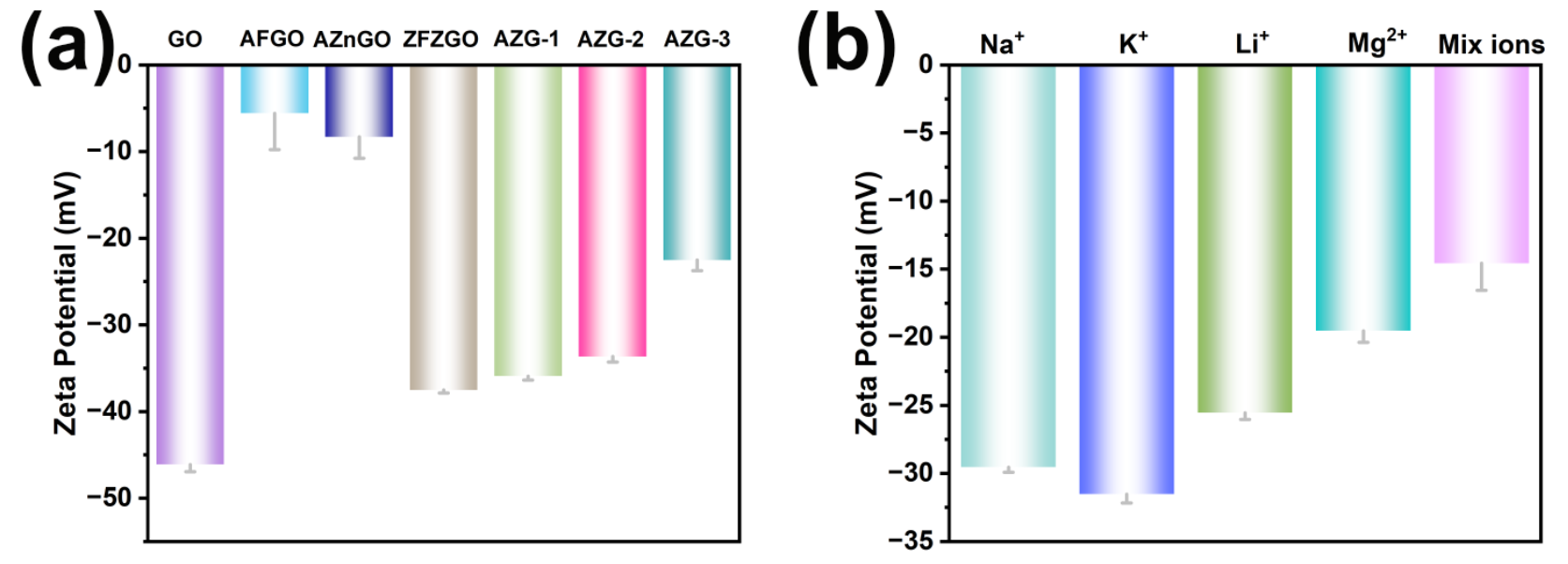


**Fig. S26.** (a) Surface zeta potential of the GO membrane Ag@ZFZ-GO membranes measured at pH of 7. (b) Ag@ZFZ-GO membrane surface zeta potential test at pH=7 after separation of different ions


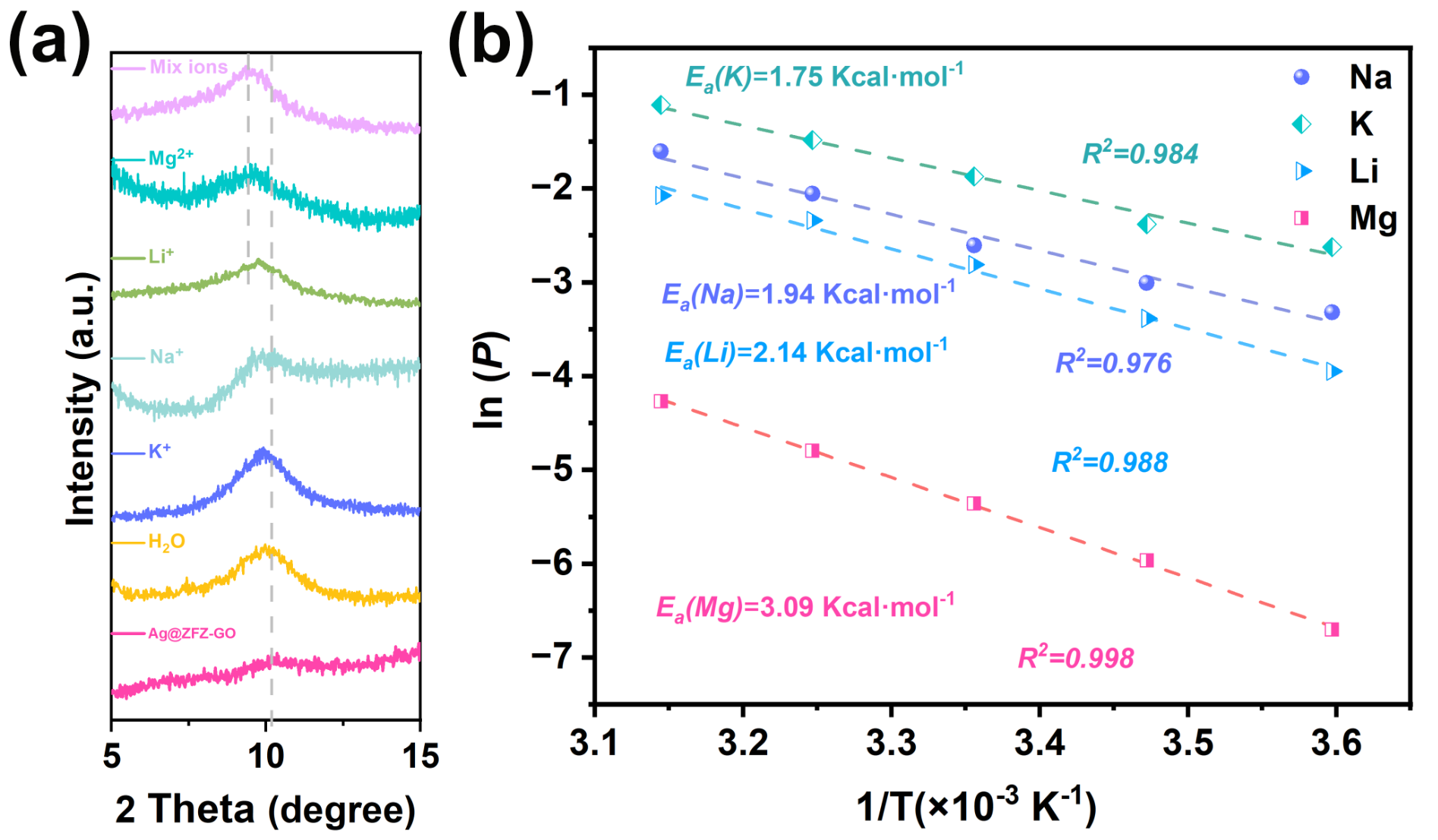


**Fig. S27.** (a) Comparison of XRD of Ag@ZFZ-GO membranes when immersed in different ionic solutions, respectively. (b) Arrhenius plots for cations diffusion through Ag@ZFZ-GO membrane in a 5×10^-3^ M mixed salts solution (KCl, NaCl, LiCl and MgCl_2_) at pH 8.0.

# Tables

**Table S1.** The comparison of the percentages for various states of O within GO, ZFZ-GO, Ag@ZnO-GO, Ag@Fe_2_O_3_-GO and series membrane materials.

| Samples | C-C | C-O | C=O |
| --- | --- | --- | --- |
| GO | 44.2% | 47.5% | 8.3% |
| ZFZ-GO | 49.7% | 36.9% | 13.4% |
| Ag@ZFZ-GO-1 | 47.9% | 37.7% | 14.4% |
| Ag@ZFZ-GO-2 | 46.5% | 39.2% | 14.3% |
| Ag@ZFZ-GO-3 | 45.9% | 41.2% | 12.9% |
| Ag@ZnO-GO | 66.1% | 26.3% | 7.6% |
| Ag@Fe_2_O_3_-GO | 46.5% | 43.8% | 9.7% |

**Table S2.** The comparison of the percentages for various states of O within ZnO, Fe_2_O_3_ and ZnFe_2_O_4_-ZnO.

| Samples | O_L_ | O_V_ | O_C_ |
| --- | --- | --- | --- |
| ZnO | 64.1% | 35.9% | - |
| Fe_2_O_3_ | 69.2% | 20.9% | 9.9% |
| ZnFe_2_O_4_-ZnO | 79.8% | 10.7% | 9.5% |

O_L_ = Lattice Oxygen.

O_V_ = Oxygen Vacancy.

O_C_ = Chemisorbed Oxygen.

**Table S3.** The comparison of the percentages for various states of O within GO, ZFZ-GO, Ag@ZnO-GO, Ag@Fe_2_O_3_-GO and Ag@ZFZ-GO series membrane materials.

| Samples | O_L_ | O_V_ | O_C_+O_cg_ |
| --- | --- | --- | --- |
| GO | - | - | 100% |
| ZFZ-GO | 47.5% | 35.1% | 17.4% |
| Ag@ZFZ-GO-1 | 45.2% | 32.9% | 21.5% |
| Ag@ZFZ-GO-2 | 35.7% | 32.6% | 31.7% |
| Ag@ZFZ-GO-3 | 28.5% | 30.1% | 41.4% |
| Ag@ZnO-GO | 35.9% | 7.5% | 56.6% |
| Ag@Fe_2_O_3_-GO | 25.1% | 19.3% | 55.6% |

O_L_ = Lattice Oxygen.

O_V_ = Oxygen Vacancy.

O_C_ = Chemisorbed Oxygen.

O_cg_= Oxygen-containing groups (e.g.,C-O and COOH)

**Table S4.** Concentration of each ion at equal ion concentration solution

| K^+^ | Na^+^ | Li^+^ | Mg^2+^ |
| --- | --- | --- | --- |
| 5×10^-3^ mol-L^-1^ | 5×10^-3^ mol-L^-1^ | 5×10^-3^ mol-L^-1^ | 5×10^-3^ mol-L^-1^ |

**Table S5.** Concentrations of individual ions in solutions of Mg-spiked ionic solutions.

| K^+^ | Na^+^ | Li^+^ | Mg^2+^ |
| --- | --- | --- | --- |
| 5×10^-3^ mol-L^-1^ | 5×10^-3^ mol-L^-1^ | 5×10^-3^ mol-L^-1^ | 2.5×10^-1^ mol-L^-1^ |

**Table S6.** Concentrations of individual ions in solutions of simulated and real salt lakes (Jie Ze Chaka Salt Lake, Tibet, China)

| K^+^ | Na^+^ | Li^+^ | Mg^2+^ |
| --- | --- | --- | --- |
| 2.61 g/L | 44.24 g/L | 0.19 g/L | 0.36 g/L |

**Table S7**. Comparison of the separation performance of the prepared 2D GO membranes with currently published ion separation membranes.

| Material | Feed solution | Water flux  (L m^-2^ h^-1^ bar^-1^) | Separation effect | Refs |
| --- | --- | --- | --- | --- |
| ZFZ-GO | Real Salt Lake | 8.30 | *SF*_(Na_^+^_/Mg_^2+^_) =_ 218.5  *SF*_(Li_^+^_/Mg_^2+^_) =_ 48.9  *SF*_(K_^+^_/Mg_^2+^_) =_ 21.5 | **This work** |
| Ag@ZFZ-GO-1 | Real Salt Lake | 32.04 | *SF*_(Na_^+^_/Mg_^2+^_) =_ 193.7  *SF*_(Li_^+^_/Mg_^2+^_) =_ 43.2  *SF*_(K_^+^_/Mg_^2+^_) =_ 77.5 | **This work** |
| Ag@ZFZ-GO-2 | Real Salt Lake | 44.37 | *SF*_(Na_^+^_/Mg_^2+^_) =_ 178.2  *SF*_(Li_^+^_/Mg_^2+^_) =_ 59.3  *SF*_(K_^+^_/Mg_^2+^_) =_ 89.1 | **This work** |
| Ag@ZFZ-GO-3 | Real Salt Lake | 37.30 | *SF*_(Na_^+^_/Mg_^2+^_) =_ 21.7  *SF*_(Li_^+^_/Mg_^2+^_) =_ 3.4  *SF*_(K_^+^_/Mg_^2+^_) =_ 8.6 | **This work** |
| Ag@ZnO-GO | Real Salt Lake | 4.51 | *SF*_(Na_^+^_/Mg_^2+^_) =_ 2.5  *SF*_(Li_^+^_/Mg_^2+^_) =_ 1.6  *SF*_(K_^+^_/Mg_^2+^_) =_ 1.75 | **This work** |
| Ag@Fe_2_O_3_-GO | Real Salt Lake | 11.01 | *SF*_(Na_^+^_/Mg_^2+^_) =_ 5.6  *SF*_(Li_^+^_/Mg_^2+^_) =_ 2.1  *SF*_(K_^+^_/Mg_^2+^_) =_ 3.97 | **This work** |
| GO | Real Salt Lake | 2.47 | *SF*_(Na_^+^_/Mg_^2+^_) =_ 2.4  *SF*_(Li_^+^_/Mg_^2+^_) =_ 1.3  *SF*_(K_^+^_/Mg_^2+^_) =_ 1.5 | **This work** |
| ZFZ-GO | Ionic solutions | 8.30 | *SF*_(Na_^+^_/Mg_^2+^_) =_ 79.5  *SF*_(Li_^+^_/Mg_^2+^_) =_ 55.4  *SF*_(K_^+^_/Mg_^2+^_) =_ 154.5 | **This work** |
| Ag@ZFZ-GO-2 | Ionic solutions | 44.37 | *SF*_(Na_^+^_/Mg_^2+^_) =_ 67.2  *SF*_(Li_^+^_/Mg_^2+^_) =_ 32.5  *SF*_(K_^+^_/Mg_^2+^_) =_ 123.7 | **This work** |
| Ag@ZFZ-GO-2 | Simulated Salt Lake | 44.37 | *SF*_(Na_^+^_/Mg_^2+^_) =_ 149.1  *SF*_(Li_^+^_/Mg_^2+^_) =_ 33.1  *SF*_(K_^+^_/Mg_^2+^_) =_ 78.4 | **This work** |
| Ag@ZFZ-GO-2 | Ionic solutions | 44.37 | *R_mg2+_=98.39%* | **This work** |
| Ag@ZFZ-GO-2 | Real Salt Lake | 44.37 | *R_mg2+_=99.21%* | **This work** |
| FGOM | Binary system  (0.1 M；1:1) | 42.78 | *SF*_(Na_^+^_/Mg_^2+^_) =_ 14.4  *SF*_(K_^+^_/Mg_^2+^_) =_ 28.3 | **^[18]^** |
| GO-Cu-24 | K^+^/Mg^2+^/Cr^3+^  (0.1 M；1:1:1) | - | *SF*_(K_^+^_/Mg_^2+^_) =_ 68.8 | **^[19]^** |
| GOs | Real Jieze Chaka Salt Lake | 17.87 | *SF*_(Na_^+^_/Mg_^2+^_) =_ 96.5  *SF*_(Li_^+^_/Mg_^2+^_) =_68.02  *SF*_(K_^+^_/Mg_^2+^_) =_ 379.17 | **^[2]^** |
| GO@PAN | Na_2_SO_4_/NaCl  (1:1) | 8.2 | *R*_Na2SO4_ = 56.7%  *R*_NaCl_ = 9.8% | **^[20]^** |
| iGO | KCl/MgCl_2_  (0.25M；1:1) | 20 | *SF*_(K_^+^_/Mg_^2+^_) =_ 9.11 | **^[21]^** |
| GO-OCMC/PSf | Mono-ionic system | 1.79 | *R*_Na2SO4_ = 92.9%  *R*_NaCl_ = 62 % | **^[22]^** |
| TA-GO | K^+^/Mg^2+^/Ni^2+^/Ca^2+^  (0.2M；1:1:1:1) | 15.4 | *SF*_(K_^+^_/Mg_^2+^_) =_ 13.90 | **^[23]^** |
| GO-PEI | K^+^/Mg^2+^/Li^+^/Na^+^  (1.0M；1:1:1:1) | - | *SF*_(Na_^+^_/Mg_^2+^_) =_ 27.0  *SF*_(Li_^+^_/Mg_^2+^_) =_ 21.9  *SF*_(K_^+^_/Mg_^2+^_) =_ 33.8 | **^[24]^** |
| (PES-GO)/PEI/TMC | Li^+^/Mg^2+^  (1:20) | 11.15 | *SF*_(Li_^+^_/Mg_^2+^_) =_ 16.12 | **^[25]^** |
| GCN-SA | Na^+^/Mg^2+^  (1.0M；1:1) | 104 | *SF*_(Na_^+^_/Mg_^2+^_) =_ 1.5 | **^[26]^** |
| GONS2-6 | K^+^/Mg^2+^/Li^+^/Na^+^  (0.2M；1:1:1:1) | - | *SF*_(Na_^+^_/Mg_^2+^_) =_ 15.3  *SF*_(Li_^+^_/Mg_^2+^_) =_ 10.7  *SF*_(K_^+^_/Mg_^2+^_) =_ 18.3 | **^[6]^** |
| SWCNT/PES | Li^+^/Mg^2+^  (1:13.9) | 12 | *SF*_(Li_^+^_/Mg_^2+^_) =_ 34.6 | **^[27]^** |
| PEI@15C5-TMC | Li^+^/Mg^2+^  (1:20) | 8 | *SF*_(Li_^+^_/Mg_^2+^_) =_ 14.0 | **^[28]^** |
| N-CPTC–TAEA | Li^+^/Mg^2+^(1:30.6)  Na^+^/Mg^2+^(2:1) | - | *SF*_(Li_^+^_/Mg_^2+^_) =_ 28.3  *SF*_(Na_^+^_/Mg_^2+^_) =_ 48.25 | **^[29]^** |
| GQDs-NH_2_ | Li^+^/Mg^2+^  (1:20) | 11.94 | *SF*_(Li_^+^_/Mg_^2+^_) =_ 27.85 | **^[30]^** |
| NFX  NF90  NF270 | Li^+^/Mg^2+^  (1:20) | 2.1  3.3  19.7 | *SF*_(Li_^+^_/Mg_^2+^_) =_ 46.4  *SF*_(Li_^+^_/Mg_^2+^_) =_ 38.3  *SF*_(Li_^+^_/Mg_^2+^_) =_ 8.5 | **^[31]^** |
| PEI-CC/PES | Seawater  (pH=8.0±0.4) | 2.4 | *SF*_(Na_^+^_/Mg_^2+^_) =_ 1.35  *SF*_(K_^+^_/Mg_^2+^_) =_ 1.39 | **^[5]^** |
| PEI/PIP-TMC | Li^+^/Mg^2+^  (1:30) | 10.6 | *SF*_(Li_^+^_/Mg_^2+^_) =_ 18.26 | **^[32]^** |
| GO/tpy-COOH-Ba^2+^  GO/tpy-COOH-Zn^2+^  GO/tpy-COOH-Cd^2+^ | Mono-ionic system  (0.5M) | 0.78  0.49  0.54 | *SF*_(Na_^+^_/Mg_^2+^_) =_ 23.5  *SF*_(Na_^+^_/Mg_^2+^_) =_ 19.5  *SF*_(Na_^+^_/Mg_^2+^_) =_ 21.4 | **^[33]^** |
| CMX-PEM | Na^+^/Mg^2+^  (4.0M；1:1) | - | *SF*_(Na_^+^_/Mg_^2+^_) =_ 2.8 | **^[34]^** |
| i-CMP | Mono-ionic system  (10mM) | - | *SF*_(K_^+^_/Mg_^2+^_) =_ 40.0  *SF*_(Li_^+^_/Mg_^2+^_) =_ 17.0 | **^[35]^** |
| M-CEM | Na^+^/Li^+^//Mg^2^  (0.1M；1:1:1:1) | - | *SF*_(Li_^+^_/Mg_^2+^_) =_ 14.1  *SF*_(Na_^+^_/Mg_^2+^_) =_ 145.7 | **^[36]^** |
| PEI | Mono-ionic system  (2000ppm) | 18.5 | *SF*_(Li_^+^_/Mg_^2+^_) =_ 21.9  *SF*_(Na_^+^_/Mg_^2+^_) =_ 20.3 | **^[37]^** |
| GO(120) NFM | Mono-ionic system  (0.02M) | 20.23 | *SF*_(Na_^+^_/Mg_^2+^_) =_ 1.17 | **^[38]^** |
| HSO_3_-UiO-66@QPPO | Synth0etic East-Taijiner salt-lake brine | 0.238 | *SF*_(Li_^+^_/Mg_^2+^_) =_ 5.92 | **^[39]^** |
| HSO_3_-UiO-66@PVC | Mono-ionic system  (1.0M) | - | *SF*_(Na_^+^_/Mg_^2+^_) =_ 5.71  *SF*_(Li_^+^_/Mg_^2+^_) =_ 4.73  *SF*_(K_^+^_/Mg_^2+^_)_ = 7.64 | **^[40]^** |
| UiO-67 | Li^+^/Mg^2^  (0.5M；1:1) | 7.22 | *SF*_(Li_^+^_/Mg_^2+^_) =_ 81 | **^[41]^** |
| UiO-66-SO_3_H | Binary system  (0.1M；1:1) | - | *SF*_(Na_^+^_/Mg_^2+^_) =_ 170  *SF*_(Li_^+^_/Mg_^2+^_) =_ 1.88  *SF*_(K_^+^_/Mg_^2+^_)_ = 5.31 | **^[42]^** |
| ZIF-8@MLDH | Binary system  (0.2M；1:1)) | ＞80 | *SF*_(Li_^+^_/Mg_^2+^_) =_ 31.9 | **^[43]^** |
| UiO-66-(COOH)_2_ | Synthetic Qinghai Taijiner salt lake | - | *SF*_(Li_^+^_/Mg_^2+^_) =_ 90.8 | **^[44]^** |
| UiO-66(Zr/Ti)–NH_2_ | Mono-ionic system | - | *SF*_(Na_^+^_/Mg_^2+^_) =_ 13.44  *SF*_(Li_^+^_/Mg_^2+^_) =_ 11.38 | **^[45]^** |
| G/Z/M | K^+^/Mg^2+^/Li^+^/Na^+^  (1:1:1:1) | - | *SF*_(Li_^+^_/Mg_^2+^_) =_ 9.5 | **^[46]^** |
| PVA/SPES/Zn-TCPP | Li^+^//Mg^2^  (0.1M；1:1) | - | *SF*_(Li_^+^_/Mg_^2+^_) =_ 8.99 | **^[47]^** |

# References

[1] W. S. Hummers, R. E. Offeman, *J. Am. Chem. Soc.* **1958**, *80*, 1339.

[2] T. Liu, X. Zhang, J. Liang, W. Liang, W. Qi, L. Tian, L. Qian, Z. Li, X. Chen, *Nano Lett.* **2023**, *23*, 9641.

[3] Z. Wang, L. Huang, X. Dong, T. Wu, Q. Qing, J. Chen, Y. Lu, C. Xu, *Nat. Commun.* **2023**, *14*, 261.

[4] J. Liang, T. Liu, Y. Li, W. Liang, X. Zhang, L. Qian, Z. Li, X. Chen, *Cell Rep. Phys. Sci.* **2022**, *3,* 100769 .

[5] K. Sharma, N. Akther, Y. Choo, P. Zhang, H. Matsuyama, H. K. Shon, G. Naidu, *Desalination* **2023**, *568*.

[6] L. Dai, S. Pang, S. Li, Z. Yi, K. Qu, Y. Wang, Y. Wu, S. Li, L. Lei, K. Huang, X. Guo, Z. Xu, *J. Membr. Sci.* **2023**, *677*.

[7] W. Xin, J. Fu, Y. Qian, L. Fu, X. Y. Kong, T. Ben, L. Jiang, L. Wen, *Nat. Commun.* **2022**, *13*, 1701.

[8] J. Liang, X. Zhang, H. Li, C. Wen, L. Tian, X. Chen, Z. Li, *Adv. Mater* **2024**, *36*, 2404629.

[9] R. Xu, Y. Kang, W. Zhang, B. Pan, X. Zhang, *Nat. Commun.* **2023**, *14*, 4907.

[10] G. Kresse, D. Joubert, *Phys. Rev. B.*  **1999**, *59*, 1758.

[11] J. P. Perdew, M. Ernzerhof, K. Burke, *J. Chem. Phys.***1996**, *105*, 9982.

[12] J. P. Perdew, K. Burke, M. Ernzerhof, *Phys. Rev. Lett.* **1996**, *77*, 3865.

[13] P. E. Blöchl, *Phys. Rev. B* **1994**, *50*, 17953.

[14] S. Grimme, J. Antony, S. Ehrlich, H. Krieg, *J Chem Phys.*  **2010**, *132*.

[15] S. Grimme, J. Antony, T. Schwabe, C. Muck-Lichtenfeld, *Org. Biomol. Chem.* **2007**, *5*, 741.

[16] L. Huang, H. Wu, L. Ding, J. Caro, H. Wang, *Angew Chem Int Ed Engl* **2024**, *63*, e202314638.

[17] B. Smit, *Chemical Reviews* **2008**, *108*, 4125.

[18] Y. Qian, J. Shang, D. Liu, G. Yang, X. Wang, C. Chen, L. Kou, W. Lei, *J. Am. Chem. Soc..* **2021**, *143*, 5080.

[19] X.-B. Lv, R. Xie, J.-Y. Ji, Z. Liu, X.-Y. Wen, L.-Y. Liu, J.-Q. Hu, X.-J. Ju, W. Wang, L.-Y. Chu, *ACS Applied Materials & Interfaces* **2020**, *12*, 56269.

[20] J. Q. Wang, P. Zhang, B. Liang, Y. X. Liu, T. Xu, L. F. Wang, B. Cao, K. Pan, *ACS Appl. Mater. Interfaces.* **2016**, *8*, 6211.

[21] M. Zhang, P. Zhao, P. Li, Y. Ji, G. Liu, W. Jin, *ACS Nano* **2021**, *15*, 5209.

[22] J. Wang, X. Gao, J. Wang, Y. Wei, Z. Li, C. Gao, *ACS Appl. Mater. Interfaces.* **2015**, *7*, 4381.

[23] M.-Y. Lim, Y.-S. Choi, J. Kim, K. Kim, H. Shin, J.-J. Kim, D. M. Shin, J.-C. Lee, *Journal of Membrane Science* **2017**, *521*, 1.

[24] Q. Huang, S. Liu, Y. Guo, G. Liu, W. Jin, *J. Membr. Sci*. **2022**, *645,* 1-9 .

[25] P. Xu, J. Hong, X. Qian, Z. Xu, H. Xia, Q.-Q. Ni, *Desalination* **2020**, *488*, 114522.

[26] Y. Wang, N. N. Wu, Y. Wang, H. Ma, J. X. Zhang, L. L. Xu, M. K. Albolkany, B. Liu, *Nat. Commun* **2019**, *10*, 2500.

[27] Z. Yang, W. X. Fang, Z. Y. Wang, R. L. Zhang, Y. Z. Zhu, J. Jin, *J. Membr. Sci.***2021**, *620*, 118862.

[28] H. W. Li, Y. Wang, T. Y. Li, X. K. Ren, J. X. Wang, Z. Wang, S. Zhao, *Chem. Eng. J.* **2022**, *438*, 135658.

[29] B. B. Yuan, S. H. Zhao, S. J. Xu, N. Wang, P. Hu, K. Chen, J. H. Jiang, J. B. Cui, X. Z. Zhang, M. You, Q. J. Niu, *J. Membr. Sci.* **2022**, *660*, 120839.

[30] P. Xu, J. Hong, Z. Z. Xu, H. Xia, Q. Q. Ni, *Separation and Purification Technology* **2021**, *258*.

[31] R. Wang, R. He, T. He, M. Elimelech, S. Lin, *Nat. Water* **2023**, *1*, 291.

[32] C. Guo, Y. Qian, P. Liu, Q. Zhang, X. Zeng, Z. Xu, S. Zhang, N. Li, X. Qian, F. Yu, *A ACS Appl. Mater. Interfaces* **2023**, *15*, 4814.

[33] P. Li, L. Jiang, L. Liu, P. Zhao, G. Xie, X. Xu, C. Liu, J. Jia, M. Liu, M. Zhang, *J. Membr. Sci.* **2022**, *655*, 120604.

[34] S. Sahin, J. E. Dykstra, H. Zuilhof, R. L. Zornitta, L. de Smet, *ACS Appl. Mater. Interfaces* **2020**, *12*, 34746.

[35] Z. Zhou, D. B. Shinde, D. Guo, L. Cao, R. A. Nuaimi, Y. Zhang, L. R. Enakonda, Z. Lai, *Adv. Funct. Mater.* **2021**, *32*, 2108672.

[36] W. Wang, Y. Zhang, X. Yang, H. Sun, Y. Wu, L. Shao, *Engineering* **2023**, *25*, 204.

[37] M. Wang, M. Li, Z. Fei, J. Li, Z. Ren, Y. Hou, *Desalination* **2022**, *544*, 116131.

[38] L. Chen, J.-H. Moon, X. Ma, L. Zhang, Q. Chen, L. Chen, R. Peng, P. Si, J. Feng, Y. Li, J. Lou, L. Ci, *Carbon* **2018**, *130*, 487.

[39] X. J. Zeng, L. Xu, T. Deng, Y. X. Wang, W. Xu, W. Zhang, *ACS. Sustain. Chem. Eng.* **2023**, *11*, 12877.

[40] C. Zhang, Y. Mu, W. Zhang, S. Zhao, Y. Wang, *J. Membr. Sci.* **2020**, *596*, 117724.

[41] R. Xu, Y. Kang, W. Zhang, X. Zhang, B. Pan, *Angew. Chem. Int. Ed.* **2021**, *61*, 202115443.

[42] T. Xu, M. A. Shehzad, X. Wang, B. Wu, L. Ge, T. Xu, *Nano-Micro Lett.* **2020**, *12*, 51.

[43] Y. Lu, R. Zhou, N. Wang, Y. Yang, Z. Zheng, M. Zhang, Q.-F. An, J. Yuan, *Nano-Micro Lett.* **2023**, *15*,147.

[44] H. Xiao, M. Chai, M. Abdollahzadeh, H. Ahmadi, V. Chen, D. B. Gore, M. Asadnia, A. Razmjou, *Desalination* **2022**, *532*.

[45] T. Xu, F. Sheng, B. Wu, M. A. Shehzad, A. Yasmin, X. Wang, Y. He, L. Ge, X. Zheng, T. Xu, *J. Membr. Sci.* **2020**, *615*, 118608.

[46] H. Liu, X. Zhang, Z. Lv, F. Wei, Q. Liang, L. Qian, Z. Li, X. Chen, W. Wu, *JACS Au* **2023**, *3*, 3089.

[47] L. Tao, X. J. Wang, F. D. Wu, B. H. Wang, C. J. Gao, X. L. Gao, *Sep. Purif. Technol.* **2022**, *296*, 121309.

1. **Correspondence. Email: liz@lzu.edu.cn (Z.L.), zx@lzu.edu.cn (X.Z.)* [↑](#footnote-ref-1)
